# The histone chaperone Vps75 forms multiple oligomeric assemblies capable of mediating exchange between histone H3–H4 tetramers and Asf1–H3–H4 complexes

**DOI:** 10.1093/nar/gkw209

**Published:** 2016-04-01

**Authors:** Colin M. Hammond, Ramasubramanian Sundaramoorthy, Mark Larance, Angus Lamond, Michael A. Stevens, Hassane El-Mkami, David G. Norman, Tom Owen-Hughes

**Affiliations:** 1 Centre for Gene Regulation and Expression, School of Life Sciences, University of Dundee, Dundee DD1 5EH, UK; 2 Nucleic Acids Structure Research Group, University of Dundee, Dundee DD1 5EH, UK; 3 School of Physics and Astronomy, University of St Andrews, St Andrews, KY16 9SS, UK

## Abstract

Vps75 is a histone chaperone that has been historically characterized as homodimer by X-ray crystallography. In this study, we present a crystal structure containing two related tetrameric forms of Vps75 within the crystal lattice. We show Vps75 associates with histones in multiple oligomers. In the presence of equimolar H3–H4 and Vps75, the major species is a reconfigured Vps75 tetramer bound to a histone H3–H4 tetramer. However, in the presence of excess histones, a Vps75 dimer bound to a histone H3–H4 tetramer predominates. We show the Vps75–H3–H4 interaction is compatible with the histone chaperone Asf1 and deduce a structural model of the Vps75–Asf1-H3–H4 (VAH) co-chaperone complex using the Pulsed Electron-electron Double Resonance (PELDOR) technique and cross-linking MS/MS distance restraints. The model provides a molecular basis for the involvement of both Vps75 and Asf1 in Rtt109 catalysed histone H3 K9 acetylation. In the absence of Asf1 this model can be used to generate a complex consisting of a reconfigured Vps75 tetramer bound to a H3–H4 tetramer. This provides a structural explanation for many of the complexes detected biochemically and illustrates the ability of Vps75 to interact with dimeric or tetrameric H3–H4 using the same interaction surface.

## INTRODUCTION

Vacuolar Protein Sorting 75 (Vps75) is a histone chaperone belonging to the Nucleosome Assembly Protein (NAP-1) family (1). The founding member of this family, Nap1 was originally identified from cell extracts as a factor capable of assembling nucleosomes *in vitro* (2). Both Nap1 and Vps75 are capable of binding all core histones H2A, H2B, H3 and H4 (1,3). However, Vps75 is best known for its ability to stimulate the histone H3 acetyl transferase activity of Regulator of Ty1 Transposition 109 (Rtt109) in *Saccharomyces cerevisiae* (4). All crystal structures published to date show Nap1 (5,6) and Vps75 (7–9) to be homo-dimeric proteins adopting characteristic ‘headphone’ folds, in which a long N-terminal helix self-associates in an antiparallel manner to form the dimerization interface which is capped at either end by a globular domain. Vps75 has also been characterized with its binding partner Rtt109 in a 2:1 and 2:2 stoichiometry (10–12), in both structures Vps75 is homo-dimeric with either one or both of the globular domains engaging an Rtt109 monomer. In addition to the headphone fold, both Nap1 and Vps75 contain C-terminal acidic domains dispensable for histone binding (9,13).

While there is excellent structural information for NAP-1 fold histone chaperones, less is known about their mode of interaction with histones. Studies of the histone:chaperone stoichiometry within these complexes indicates that both Vps75 and Nap1 dimers associate with two histone H3–H4 or H2A–H2B dimers (9,14). Subsequently, Nap1 and Vps75 were found to associate with histone H3 and H4 in a tetrameric nucleosomal-like configuration (15). A recent study provided evidence for a Nap1 dimer binding an unconventional tetrameric assembly of H2A and H2B (16). In contrast to these studies, other studies have reported that a dimer of Nap1 binds a histone fold dimer (13,17). Thus there is a certain level of dispute as to nature and stoichiometry of NAP-1 fold chaperones in complex with their histone cargo. In the light of the recent observations that Vps75 and Nap1 form homo-tetramers in solution under physiological-like salt concentrations (18), the assumption that a NAP-1 fold dimer recognizes a H3–H4 tetramer may also be flawed. However, the relevance of the tetrameric form of these chaperones with respect to histone binding has yet to be fully addressed; indeed the tetrameric Vps75 ring does not have dimensions capable of accommodating a histone tetramer (18).

In addition to Vps75, the histone chaperone Anti-Silencing Function 1 (Asf1) is also able to stimulate the catalytic activity of Rtt109 (4). In contrast to Vps75, Asf1 binds a dimer of H3–H4 and in doing so occludes the histone tetramerization interface (19,20). The two chaperones also direct the catalytic activity of Rtt109 towards distinct sites on histone H3. Asf1 is required for the acetylation of H3 K56 by Rtt109 *in vivo* (21,22), whilst Vps75 promotes Rtt109 catalysed acetylation of H3 K9 and K27, sites which are also redundantly acetylated by Gcn5 (23,24). Thus Vps75 and Asf1 display similarities in the ability to bind H3–H4 and stimulate Rtt109 activity, and differences in their mode of interaction with H3–H4 and their direction of Rtt109 specificity. With the aim of providing new insight into these functions, we sought to further characterize the interaction of Vps75 with histones H3 and H4.

## MATERIALS AND METHODS

### Protein expression and purification

Vps75, Nap1, Asf1g (residues 1–164) and H3–H4 were expressed and purified as reported previously (15). Vps75 Y35C was expressed in Spectra9 deuterated media (Cambridge Isotope Laboratories) for PELDOR experiments. The VAH complex was reconstituted by mixing equimolar amounts of Vps75 dimers with Asf1g monomers to 14 ml of buffer, as indicated in sections below, and finally slowly adding one equivalent of H3–H4 dimer prior to concentrating the sample in an Amicon Ultra-15 ml 10 kDa MWCO centrifugal filter to >1 ml and purifying the complex in a pre-equilibrated a Superdex 200 column.

### Crystallization and X-ray data collection

Crystals of the Vps75 tetramer were obtained via sitting drop vapour diffusion by mixing, in a 1:1 ratio, the Vps75–Asf1-H3–H4 (VAH) protein complex with the well solution of 0.1 M sodium cacodylate pH 6.5, 25% (w/v) PEG 4000 as part of the 96 well Protein Complex Suite (Qiagen) crystallization screen. The VAH complex was prepared by sequentially adding 6 nmol of purified Vps75 (1–225) dimer, Asf1 (1–164) monomer and H3–H4 dimer to ∼12 ml of 400 mM NaCl, 30 mM HEPES–KOH pH 7.5 and 30% glycerol. Following incubation for 30 min at room temperature the complex was concentrated to ∼1.5 ml and purified on a HiLoad 16/600 Superdex 200 column (GE Healthcare) pre-equilibrated in the aforementioned buffer. The VAH complex was seen to elute at ∼69 ml, dimeric Vps75 (1–225) eluted at ∼72 ml and Asf1 (1–164) eluted at ∼92 ml. Fractions spanning the peak elution volume were pooled and concentrated, finally the glycerol was diluted to 5% prior to concentrating the complex for crystallization. Crystals took 3 months to grow and were cryo-protected with 50% glycerol prior to X-ray diffraction collection. Diffraction data was collected at −173°C on beamline I04 at Diamond Light Source (Oxford, UK) equipped with a Pilatus 6M-F (Dectris) detector and ACTOR sample changer (Rigaku). The crystal data statistics are provided in Table 1. Data collected was indexed and integrated using iMosFLM and scaled with SCALA, both as part of the CCP4 program suite (25).

**Table 1.** Crystal data processing and refinement statistics

Protein Data Bank Code	5AGC
Space group	*C* 1 2 1
Unit Cell Dimensions	
*a, b, c* (Å)	87.75, 92.43, 160.75
*α, β, γ* (°)	90.00, 93.85, 90.00
Resolution range (Å)	50.0–4.0
Unique reflections / Redundancy	9771/5.1
Completeness (%)	98.9
<*I*/*σ*(*I*)> (at 4 Å)	3.4
Rsym	0.14
Wilson *B* (Å^2^)	124.4
Refinement	Refmac 5.6.00117
Total number of protein atoms	7250
*R*_work_/*R*_free_ (%)	22.9/26.5
Overall *B* factor (Å^2^)	139
RMSD bond lengths (Å)/bond angles (°)	0.009/1.4
*F*_o_, *F*_c_ correlation	0.91
Ramachandran plot (%)	
Most favored regions	95.89
Additional allowed regions	3.48
Disallowed regions	0.63

### Structure determination and refinement

**Figure 1.**
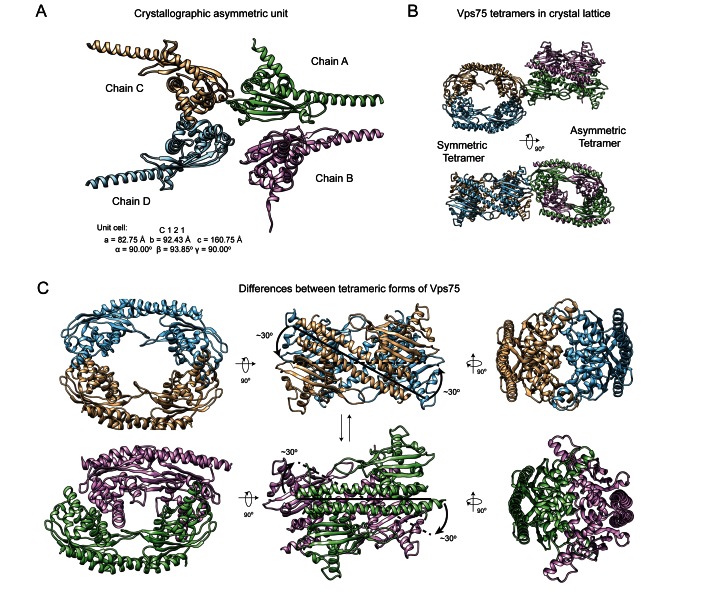
Crystallographic forms of the Vps75 tetramer. (**A**) The asymmetric unit of Vps75 crystallized in tetrameric conformations (PDB code: 5AGC). (**B**) Tetrameric assemblies of Vps75 present in the crystal lattice. Vps75 forms both a symmetrical ring-like tetramer and an asymmetrical ring like tetramer in the crystal lattice. Chains A–D are colored as in (A). (**C**) The differences between the two tetrameric forms of Vps75. An ∼30° rotation at the tetramerization interface (middle) is largely responsible for the inter-conversion between the symmetric tetramer, where the globular domains of each monomer interlock (top-right), and the asymmetric tetramer, where the globular domain of one monomer of each dimer occupies the concave groove of the opposing dimer in the Vps75 tetramer (bottom-left). For clarity each dimer of Vps75 is distinguished by color.

**Figure 2.**
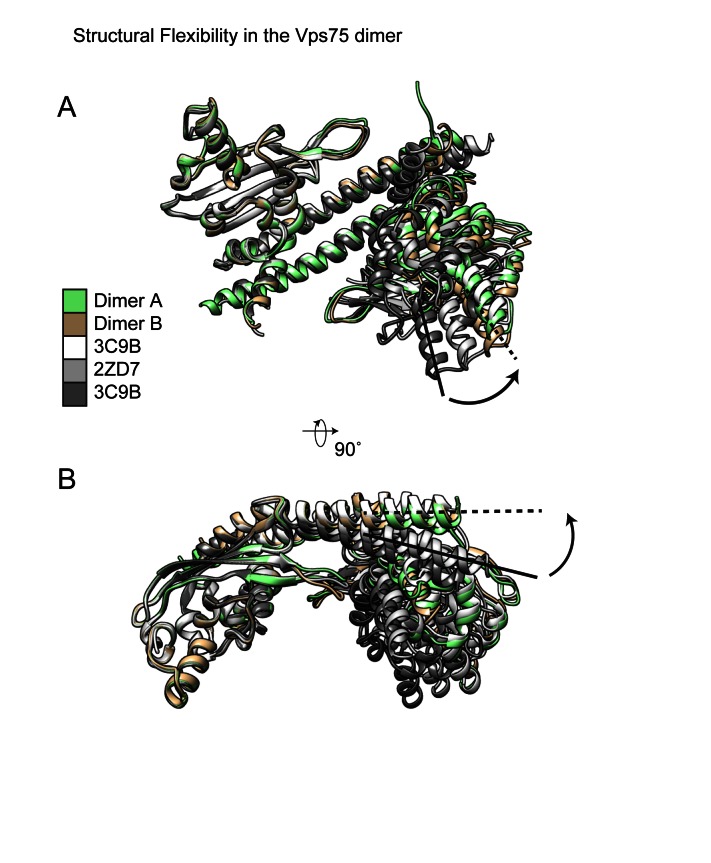
Structural flexibility in the Vps75 dimer. Vps75 dimers from the asymmetrical and symmetrical forms of the Vps75 tetramer (dimers A and B respectively) along with previously solved crystal structures (PDB codes 3C9B (8) and 2ZD7 (9)) were aligned to chain A of the Vps75 dimer 3C9D (8). (**A**) In all instances the globular domains of the aligned monomers are well aligned but significant deviations are seen in the trajectory of the long dimerization helix, (**B**) this in turn causes the volume between the two globular domains of Vps75 monomers in the dimer to vary between crystal structures. The tetrameric dimers adopt a conformation most closely matched by the previously solved Vps75 dimer structure containing selenomethionine (3C9B), with a comparatively large volume between the two globular domains in the Vps75 dimer.

The crystal structure of the tetrameric forms of Vps75 (PDB code: 5AGC) was solved via molecular replacement using PHASER (26) of the CCP4 program suite (25) with four copies of a Vps75 monomer (PDB code: 2ZD7 (9)) as the search ensemble. The solution was further refined in REFMAC5 (27) in the following manner: one round of rigid body refinement was followed by several rounds of restrained jelly-body refinement (sigma = 0.02) against local, automatically generated NCS restraints. There are four Vps75 monomers in the asymmetric unit, which produce two distinct tetrameric structures in the crystal lattice as described in the text. Model building was performed in COOT (28). The geometry of the final model was assessed using MolProbity (29) prior to deposition to the Protein Data Bank (30) using the AutoDep server of PDBe (31). Figures 1 and 2 were produced using Chimera v1.9 (32).

### Amine reactive cross linking

BS2G, BS3 and DTSSP cross-linkers (Thermo Scientific) were made as 50 mM stock solutions in DMSO and stored at −20°C for up to 1 week. All assays were performed with versions of yeast Vps75, Asf1 and Xenopus histones H3 and H4 from which native cysteine residues had been removed by mutagenesis. For cross-linking titrations the components, were assembled as described in figure legends in 20 mM HEPES pH 7.5 with concentrations of NaCl as indicated. After incubating the mixtures at room temperature for 30 min the samples were cross-linked with 2 mM BS2G for 1 h and quenched with 50 mM ammonium carbonate prior to SDS-PAGE analysis. Cross-linking titrations with Vps75 and H3–H4 were performed twice and representative gels are shown in Figure 4.

For DTSSP cross-linking prior to SEC-MALS analysis—Vps75 dimers were mixed with H3–H4 dimers at a final concentration of 10 μM in 200 mM sodium chloride and 20 mM HEPES–KOH to a final volume of 15 ml. Following equilibration at room temperature for 30 min 2 μl of DTSSP (50 mM) was added and cross-linking was allowed to proceed for 30 min before quenching the reaction with 10 μl with ammonium carbonate (1 M). Any precipitate was removed via centrifugation and the supernatant was concentrated to <100 μl prior to SEC-MALS analysis in 400 mM sodium chloride, 20 mM HEPES–KOH pH 7.5. SEC-MALS analysis was repeated at least once to ensure all observations were reproducible.

For MS/MS analysis of cross-linked peptides: 7.5 nmol C-terminally truncated Vps75 (Vps75g residues 1–225), Asf1g (residues 1–164) and histone H3–H4 dimers were each combined at 0.5 uM in 400 mM sodium chloride, 30 mM HEPES–KOH. Low protein concentrations were used to favor cross-links within the complex over cross-links between complexes. The mixture was allowed to equilibrate for 30 min at room temperature prior to cross-linking for 1 h with 20 molar equivalents of isotopically labeled BS2G (d0/d4). Unreacted NHS-esters were quenched using 50 mM ammonium carbonate. The cross-linked complex was then concentrated to 10 uM and purified via gel filtration on a Dionex Ultimate 3000 HPLC system using a Superdex 200 10/300 GL column (GE Healthcare) equilibrated with 400 mM sodium chloride, 30 mM HEPES–KOH pH 7.5. The complex eluted from the column as a single peak and highly cross-linked species were enriched by SDS-PAGE isolation or denaturing gel filtration in 6 M guanidinium HCl, 100 mM Tris–HCl pH 8.0 across two experiments. For in solution digests fractions enriched with highly cross-linked species were combined and buffer exchanged with 1 M urea, 100 mM Tris–HCl pH 8.0. Standard in gel and in solution digest protocols were followed using a 1:50 w/w ratio of sequencing grade trypsin (Roche) overnight at 37ºC.

### Sulfhydryl-reactive crosslinking

To trap H3–H4 in a tetrameric conformation, H3 K115C/H4 tetramers were reconstituted and cross-linked with a 1:1 ratio of bis-maleimidoethane (BMOE) as reported previously (15).

### Pyrene excimer studies

Following size exclusion chromatography, fractions spanning the eluted peak of Vps75 K78C were pooled and concentrated to ∼1 ml using a 10 kDa MWCO Amicon Ultra centrifugal filter (Millipore). To the concentrated protein 1–2 mg of N1-(pyrene)maleimide (Sigma Aldrich) was added as a powder, the reaction was allowed to proceed overnight with agitation at 4°C. The following day precipitate was removed by centrifugation and size exclusion chromatography was performed to remove unreacted N1-(pyrene)maleimide and soluble aggregates. The eluted protein was concentrated and the concentration was calculated spectrophotometrically using the protein extinction coefficient of a Vps75 dimer 90,760 M^−1^ cm^−1^ at 280 nm. Pyrene fluorescence experiments were performed with two repeats on a Cary Eclipse Fluorescence Spectrophotometer (Varian) with the parameters summarized in Supplementary Table S1. Protein and BS3 cross-linker concentrations for Pyrene excimer experiments are stated in the legend to Figure 5.

### Analytical gel filtration

Analytical gel filtration experiments were performed on an Akta purifier or Dionex Ultimate 3000 HPLC system using a 120 ml HiLoad 16/60 Superdex 200 (GE Healthcare – Figure 6) or 2.4 ml Superdex 200 PC 3.2/30 (GE Healthcare – Supplementary Figure S5) column equilibrated with 400 mM sodium chloride, 20 mM HEPES–KOH pH 7.5 and 30% glycerol. A total of 31 × 2 ml fractions were collected spanning the void to bed volume of the HiLoad 16/60 Superdex 200 column from 41 to 103 ml. A total of 24 × 80 μl fractions were collected spanning the void to bed volume of the Superdex 200 PC 3.2/30 column from 0.48 to 2.40 ml, a maximum of 6 nmol of total protein in less than 25 μl was injected per Superdex 200 PC 3.2/30 run. Fractions were analysed by SDS-PAGE and numbered sequentially (Figure 6 and Supplementary Figure S5).

### PELDOR sample preparation

Histone tetramers were reconstituted and labeled with MTSL or 3,4-bis(MTSL) as described previously (18,33) and deuterated Vps75 Y35Rx2 prepared as described previously (18). The components of each EPR sample were buffer exchanged for 800 mM sodium chloride, 20 mM HEPES pH 7.5 in D_2_O and concentrated. To prevent precipitation of the histone chaperone complex the order in which proteins were mixed was found to be critically important. The chaperones were first mixed together: one equivalent of Vps75 Y35Rx2 dimers (5 nmol) was added to 1.5 equivalents of globular Asf1 monomer (residues 1–164, 7.5 nmol) and made up to 50 μl with the aforementioned buffer and D_8_-glycerol to make the final buffer conditions (400 mM sodium chloride, 20 mM HEPES–KOH pH7.5, 50% D_8_-glycerol in D_2_O). Separately, one equivalent of spin labeled H3–H4 dimer (5 or 2.5 nmol H3–H4 tetramer) was similarly made up to 50 μl with the aforementioned buffer and D_8_-glycerol. Finally the spin labeled tetramer mixture was added to the chaperone mixture, but not vice versa, and mixed rapidly by pipette.

### Pulsed electron–electron double resonance

PELDOR experiments were performed as described previously (18). Experiments were performed at X-band with the exception of the Asf1 + H3–H4(N25R1)–Vps75 Y35Rx2 sample which was run at Q-band with the same X-band Bruker Elexsys E580 spectrometer but an upgraded Traveling Wave Tube (TWT) amplifier. Data analysis was carried out as described previously (18).

### LC–MS/MS analysis of cross-linked peptides

The peptide products from trypsin digests were acidified by the addition of trifluoroacetic acid to 1% final concentration and desalted using 10 mg tC18 Sep-Pak 96-well plates according to manufacturer's instructions (Waters, Milford, MA, USA). Desalted peptides were dried using centrifugal evaporation at 40°C and resuspended in 20 μl of 5% formic acid. The peptide mixture (1 μl) was injected onto a 15 cm EasySpray C18 column (Thermo Fisher) and separated by a linear organic gradient from 2 to 35% buffer B (80% acetonitrile, 0.1% formic acid) over 110 min. Peptide ions were generated by electrospray ionization from the EasySpray source and introduced to a Q Exactive mass spectrometer (Thermo Fisher). Peptide ions of charge +3 to +6 also showing two isotopic envelopes separated by 4.0251 Da 10 parts per million (due to the isotope-labeled cross-linker), were chosen for HCD fragmentation and MS/MS analysis. Intact peptide ion and fragment ion peaks were extracted from the RAW format into MGF format using the Proteome Discoverer 1.4 software package, for subsequent analysis.

Cross-linked peptides were assigned using Hekate software (34) using a MS1 tolerance of 1 PPM, MS2 tolerance of 0.1 PPM, with charge matching between isotopically labeled BS2G-d0 and BS2G-d4 cross-linked peptides. Peptides were scored by matching B and Y-ions and a false discovery rate obtained from a decoy search was used as a cut-off for accepting annotated cross-linked peptides. Isotope matched (d0 and d4) cross-linked peptides observed >2 times with alpha and beta peptides of ≥5 residues and a false discovery rate of ≤5% were accepted for further consideration (Supplementary Table S2).

### Rigid body energy minimization with XPLOR-NIH

Crystal structures of the Vps75 dimer (PDB code: 2ZD7)(9) and Asf1 bound to histones (PDB code: 2HUE) (19) with added spin label ensembles were docked together by energy minimization experiments in XPLOR-NIH against distance restraints. These experiments were based on the docking protocol (18) used to solve the structure of the Vps75 tetramer from distance restraints (18). Briefly, R1 spin label ensembles were simulated in MTSSLwizard (35) and Rx2 spin labels in XPLOR-NIH (36). Model distance measurements between spin label ensembles and cross-linking restraints between lysine side chains (Nζ–Nζ) were input as NOE distance restraints. Starting coordinates of Vps75, Asf1H3–H4 and associated spin label ensembles were generated with random relative rotational orientations between Vps75 and Asf1H3–H4. Each set of starting coordinates were energy minimized against distance restraints using a combination of rigid-body minimization and internal coordinate space dynamics. The energy of the interaction between Vps75 and Asf1H3–H4 was evaluated, after energy minimization, for each set of coordinates. The final coordinates were either accepted, or rejected, via energy term criteria, which were set at appropriate levels as assessed from plots of the total and the NOE energy terms in ascending order of the NOE energy term. Accepted coordinates were aligned and averaged, energy terms for accepted coordinates are quoted in the VAH_aligned.pdb file and the RMSD of atoms in accepted coordinates compared to the average coordinates are quoted in the VAH_average.pdb file (see Supplementary Data).

## RESULTS

### Crystallographic forms of the Vps75 tetramer

While attempting to crystallize Vps75 in complex with histones H3–H4, we inadvertently crystallized Vps75 with four Vps75 monomers in the asymmetric unit (Figure 1A). Statistics from the X-ray diffraction data collection are summarized in Table 1. Upon repetition of the asymmetric unit, two distinct tetrameric assemblies of Vps75 are clearly observed in the crystal lattice (Figure 1B). One tetramer is made up of two dimers of Vps75, each composed of a copy of chains A and B, which together form an asymmetric ring-like tetramer. The second, symmetrical ring-like tetramer, is composed of a dimer of chain C and a dimer of chain D. Both tetramers are related structures, with the latter most closely resembling the conformation previously observed in solution (18) (Supplementary Figure S1). In the symmetrical tetramer, the antiparallel dimerization helices of one dimer form a saltire-like cross with those of the opposing dimer (top middle, Figure 1C). A ∼30° dislocation in the register of the two dimers allows the symmetrical tetramer to transition into the asymmetrical tetramer (bottom middle, Figure 1C) and *vice versa*. In the symmetrical tetramer the ring-like cavity is open at both sides (top left, Figure 1C). However, the transition to the asymmetrical tetramer is also accompanied by a closure of one side of the cavity as the globular domain of one dimer occupies the cavity of the opposing dimer and *vice versa* (bottom left, Figure 1C). The cavity on the other side of the ring remains open (not shown). Amino-acid conservation analysis shows that residues mapping to the asymmetric and symmetric tetramerization interfaces of Vps75 are well conserved (Supplementary Table S3 and Figure S2).

A previous study (8) reported significant structural deviations between a Vps75 dimer (PDB code: 3C9D, Figure 2) and a selenomethionine substituted Vps75 dimer (PDB code: 3C9B, Figure 2). An additional structure of the Vps75 dimer (PDB code: 2ZD7, Figure 2), with a conformation intermediate between the aforementioned structures has also been reported (9). Structural deviations in the Vps75 dimer have also been reported in the structures of Vps75 bound with Rtt109 in different stoichiometries (not shown) (10–12). Figure 2 shows a comparison of the Vps75 forms within the tetramer, with those observed in previous dimer structures (in the absence of Rtt109) (Figure 2). The tetrameric Vps75 dimers (dimers A and B, Figure 2) most closely resemble the selenomethionine substituted Vps75 dimer (PDB code: 3C9B, Figure 2). Thus, in the tetrameric conformation, dimers of Vps75 have a relatively large acidic concave groove between the globular domains of each Vps75 monomer (Figure 2A) as a result of a straightening of the antiparallel dimerization helices (Figure 2B).

### Vps75 binds H3–H4 in multiple oligomeric assemblies

Our previous study showed Vps75 capable of binding histone H3 and H4 in a tetrameric conformation (15), however the oligomeric state of Vps75 was not investigated in that instance. To investigate the size of Vps75–H3–H4 complexes we subjected mixtures of Vps75 and histones to size exclusion chromatography coupled to multi-angle light scattering (SEC-MALS). The mass of the major species formed was observed to decrease from ∼175 to ∼150 kDa across the elution peak (Supplementary Figure S3A), consistent with the presence of a series of complexes including tetramers of Vps75 bound to histone H3–H4 tetramers (178 kDa) and partial dissociation products thereof. The dissociation of the complex during the chromatographic separation may explain why H3–H4 was only enriched in the first few fractions analysed by SDS-PAGE (Supplementary Figure S3B).

To combat these issues and gain a better estimate of the size of the Vps75–H3–H4 complex(es) we partially cross-linked them before concentrating the complex for SEC-MALS analysis. Related cross-linking approaches have been successfully used to stabilize complexes prior to electron microscopy (37). For this analysis, we chose the reversible cross-linker 3,3′-dithiobis[sulfosuccinimidylpropionate] (DTSSP). The ability to reverse cross-links with dithiothreitol (DTT) makes it possible to assess which polypeptides are present within the complex by SDS-PAGE.

**Figure 3.**
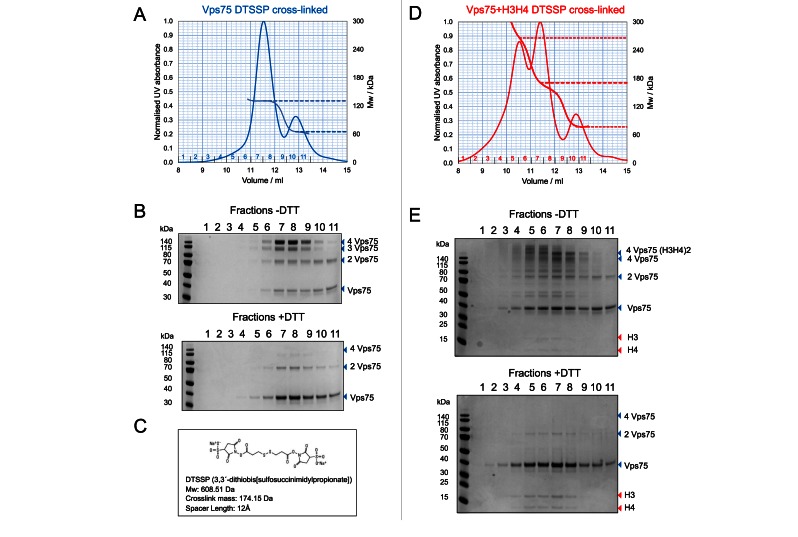
Vps75 associates with histones H3–H4 in multiple oligomeric assemblies. SEC-MALS analysis of DTSSP cross-linked Vps75 and Vps75 with histones H3–H4. (**A**) SEC-MALS analysis of Vps75 (5 nmol) cross-linked with DTSSP (100 nmol) at 200 mM NaCl, 20 mM HEPES pH 7.5 and eluted from Superdex 200 10/300 GL column (GE Healthcare) at 400 mM NaCl, 20 mM HEPES. Dashed line indicates the molecular weights at the peak elution volume peak elution volume. Fraction numbers are indicated in blue. (**B**) SDS-PAGE analysis (4–12% Bis–Tris NuPAGE gels) of fractions from the SEC-MALS analysis of cross-linked Vps75 and fractions treated with DTT. (**C**) Details of DTSSP cross-linker. (**D**) SEC-MALS analysis of Vps75 (5 nmol) and H3–H4 (5 nmol) cross-linked as described in (A) with fraction numbers are indicated in red. (**E**) SDS-PAGE analysis of fractions from the SEC-MALS analysis of cross-linked Vps75 H3–H4 as described in (B).

To test the validity of this approach Vps75 (in the absence of histones) was partially cross-linked with DTSSP at 200 mM sodium chloride—conditions that favour the Vps75 tetramer. Subsequently, the complex was concentrated and SEC-MALS analysis performed at 400 mM sodium chloride—high salt favours the Vps75 dimer (18) (Figure 3A). The analysis was performed at 400 mM sodium chloride to reduce protein losses in subsequent Vps75–H3–H4 separations and to allow direct comparison between the Vps75 and Vps75–H3–H4 SEC-MALS experiments. The SEC-MALS of Vps75 chromatogram shows a large proportion of cross-linked Vps75 tetramer (11.5 ml, 130.7 kDa) separated from residual cross-linked Vps75 dimers (12.9 ml, 65.3 kDa) (Figure 3A). The difference in the observed mass and the theoretical mass of the Vps75 dimer construct (62.6 kDa) of 2.7 kDa equates to the presence of approximately 15 cross-links (174 Da) in addition to the two polypeptides chains of Vps75. In the case of the Vps75 tetramer (125.3 kDa) this difference is 5.4 kDa which equates to approximately 31 cross-links. As there are 26 lysine residues in each chain of Vps75 it is reasonable that this level of cross-linking and or dead-end cross-linked products accounts for the difference between the observed and theoretical masses. SDS-PAGE analysis of fractions spanning the peak at 11.5 kDa shows four bands indicative of a cross-linked Vps75 tetramer that can be reduced to a predominantly monomeric species by DTT (Figure 3B).

Subsequently, Vps75 was mixed with histones H3–H4 in a one dimer to one dimer stoichiometry and again subjected to partial DTSSP cross-linking at 200 mM sodium chloride. SEC-MALS analysis of the cross-linked material (at 400 mM sodium chloride) resulted in three main peaks in the chromatogram (Figure 3D). The presence of multiple Vps75–H3–H4 species is consistent with our previous assessment that Vps75 forms multiple oligomeric assemblies with H3–H4 (Supplementary Figure S3B) and has been reported for Nap1(3,17). However, due to insufficient chromatographic separation the nature of these species could not be accurately assigned. This highlights a limitation of the SEC-MALS approach; which reports on the average mass of all species passing through the light scattering and differential refractive index detectors. Nevertheless, the mass at the centre of each eluting peak gives an approximate measure of the size of complex that may be present in solution. The smallest species is most likely that of a Vps75 dimer, because it elutes at the same volume as the cross-linked Vps75 dimer observed previously (12.9 ml, 65.3 kDa – Figure 3A). However, the mass of this species (∼77 kDa), is influenced by the adjacent eluting species (evidenced by bands above the Vps75 dimer in fraction 10, Figure 3E). The central peak and most predominant species eluting at 11.4 ml has a mass at the centre of the peak of ∼170 kDa—close to that of a Vps75 tetramer plus a tetramer of H3–H4 (178.2 kDa). The largest species eluting at 10.5 ml (∼266 kDa) could not be assigned with confidence. However, the presence of this species demonstrates the ability of the Vps75 H3–H4 complex to form high order oligomers similar to Nap1 H3–H4 complexes (3,17).

### A dimer of Vps75 binds a dimer of H3–H4 in a manner compatible with H3–H4 tetramerization

The fact that Vps75 can associate with histones H3–H4 in multiple oligomeric assemblies suggests a complex binding equilibria between Vps75 and H3–H4. To investigate these equilibria cross-linking reactions with Vps75 titrated with H3–H4 were analysed by SDS-PAGE. For this purpose, the amine reactive cross-linker Bis(sulfosuccinimidyl) glutarate (BS2G) was selected to promote cross-linking specificity due to its relatively short cross-linking radius (7.7 Å). Additionally, the reduced cross-linking radius of BS2G aided the assignment of high molecular weight cross-linked species due to the observation of intermediate cross-linked species.

**Figure 4.**
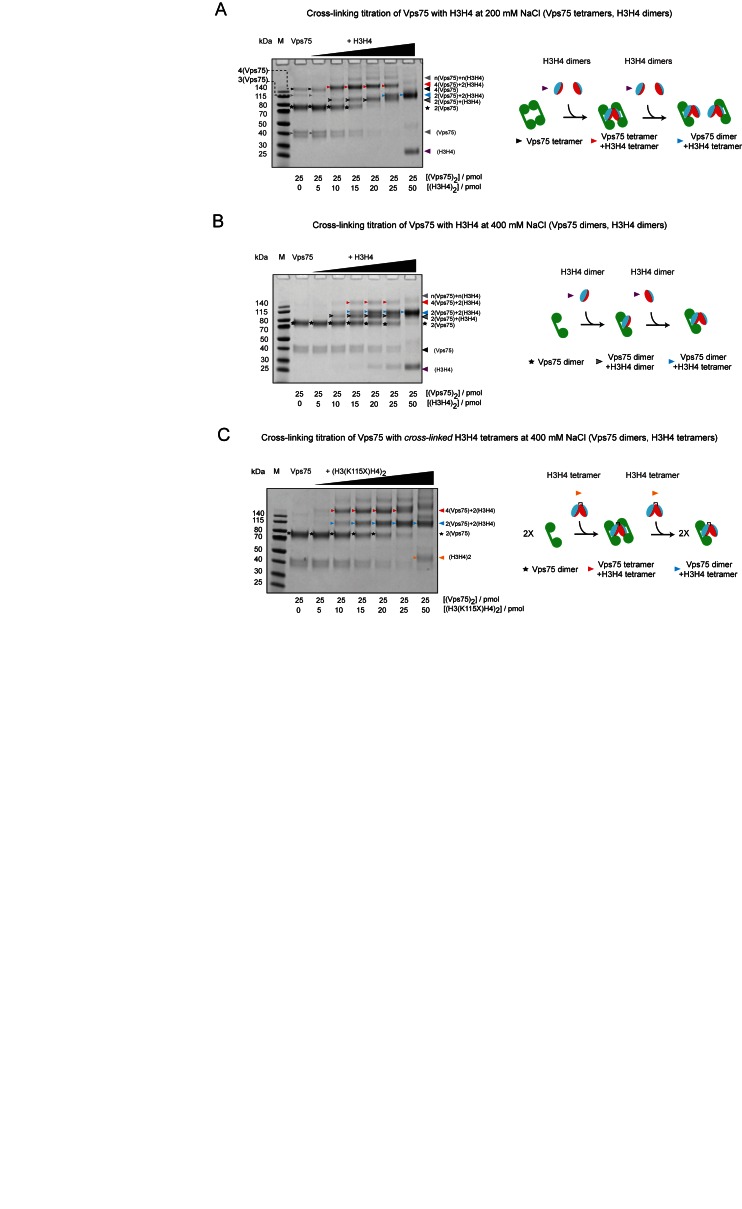
The Vps75 tetramer can cooperatively recognize a H3–H4 tetramer at low salt concentrations. SDS-PAGE analysis of cross-linking reactions of wild-type Vps75 incubated with increasing concentrations of H3–H4 at (**A**) 200 mM NaCl and (**B**) 400 mM NaCl. (**C**) SDS-PAGE analysis of cross-linking reactions of wild type Vps75 incubated with increasing concentrations of pre-cross-linked, by BMOE linkage of H3 K115C (denoted K115X), H3–H4 tetramers at 400 mM NaCl. All cross-linking reactions were performed for 1 h with 2 mM BS2G and run on 4–12% Bis–Tris SDS-PAGE gels (Invitrogen). M = PageRuler Prestained Protein Ladder (Thermo Scientific). Molar amounts of Vps75 and H3–H4 present in each titration point are shown below each lane. Bands are marked with arrows and stars as referred to in the schematic next to each titration. Higher order oligomers are observed above the Vps75 tetramer bound to a H3–H4 tetramer in titrations (A) and (C) which may indicate the presence of high order oligomers. Vps75 H3–H4 complexes observed in cross-linking titrations were interpreted as follows: (A) Under conditions that favour the Vps75 tetramer (200 mM NaCl), a cross-linking species of a Vps75 tetramer bound to a H3–H4 tetramer (red arrow) is preferred at low histone stoichiometries, at high histone stoichiometry each dimer of Vps75 is bound to a H3–H4 tetramer (blue arrow); (B) under conditions that favour a Vps75 dimer (400 mM NaCl), initially the dimer of Vps75 binds a dimer of H3–H4 (gray arrow with black outline) in a manner that does not obstruct H3–H4 tetramerization—which then occurs on Vps75 as the H3–H4 stoichiometry increases (blue arrow); (C) under conditions that favour a Vps75 dimer (400 mM NaCl) and when H3–H4 is trapped in a tetrameric conformation, initially the two dimers of Vps75 bind the preformed H3–H4 tetramer (red arrow), at high histone stoichiometry the histone binding capacity of Vps75 is saturated and each dimer binds a pre-formed H3–H4 tetramer (blue arrow).

At 200 mM sodium chloride, in the absence of histones, cross-linked Vps75 forms four distinct bands which migrate at molecular weights consistent with monomeric, dimeric, trimeric and tetrameric cross-linked Vps75 during SDS-PAGE analysis (Figure 4A). At 200 mM sodium chloride (Figure 3A) and under physiological-like salt concentrations (18), Vps75 is predominately tetrameric in solution, yet the abundance of the tetrameric BS2G cross-linked species is lower than that of the dimer (Figure 4B). Therefore cross-linking products observed by SDS-PAGE must be assessed with caution in view of these limitations. In this instance, the reduced BS2G cross-linking efficiency of the Vps75 tetramer likely reflects differences in the surface area and proximity of primary amines at the Vps75 dimer and tetramer interfaces. It is notable that tetrameric Vps75 is much more efficiently cross-linked with DTSSP at 200mM sodium chloride (Figure 3A) due to the larger cross-linking radius of DTSSP compared with BS2G. Furthermore, a cross-linked tetramer requires a minimum of three simultaneous cross-links to be retained during denaturing gel electrophoresis, compared with one cross-link required to stabilize a dimer. At 400 mM sodium chloride, the cross-linking of the Vps75 tetramer is greatly reduced, consistent with the dissociation of Vps75 tetramers under these conditions (Figure 4B and C).

The cross-linking pattern of Vps75 incubated with increasing concentrations of H3–H4 varies dependent on whether the reaction is performed at 200 or 400 mM sodium chloride, conditions that favour either tetrameric, or dimeric, Vps75 respectively. At early titration points when Vps75 is in excess of H3–H4 the most prominent cross-linked species observed at 200 mM sodium chloride is larger than the Vps75 tetramer and consistent with H3–H4 binding to the Vps75 tetramer (red arrow, Figure 4A). This species is also observed at 400 mM sodium chloride but is less prominent (red arrow, Figure 4B). In principle, this complex could result from the association of either a tetramer, or dimer, of H3–H4 with the Vps75 tetramer. However, as the same species was observed when Vps75 was bound to H3–H4 trapped in a tetrameric conformation (red arrow, Figure 4C), we deduce that this species contains tetramers of both Vps75 and H3–H4.

From these data, we interpret that formation of Vps75 tetramers bound H3–H4 tetramers is favoured at either 200mM NaCl when Vps75 alone is tetrameric (Figure 4A), or when H3–H4 is trapped in a tetrameric conformation (Figure 4C). In both instances, at higher histone ratios, when Vps75 becomes limiting, the most prominent species drops down in molecular weight to a Vps75 dimer plus a H3–H4 tetramer (blue arrow, Figure 4A and C). In contrast, at 400 mM sodium chloride, the cross-linking pattern suggests that Vps75 first binds a H3–H4 dimer and as the titration proceeds this is converted into the species interpreted as a Vps75 dimer bound to a H3–H4 tetramer (blue arrow, Figure 4B). Together these data suggest that a dimer of Vps75 binds a dimer of H3–H4 in a manner that is compatible with H3–H4 tetramerization.

This shows that both dimeric and tetrameric Vps75 are capable of binding a H3–H4 tetramer, with the species adopted in solution dependent upon the histone:chaperone stoichiometry. We propose schemes depicting our interpretation of each cross-linking titration experiment in which Vps75 dimers interact with H3–H4 dimers in a manner that does not occlude the H3–H4 tetramerization interface (Figure 4A–C). Thus, the Vps75 tetramer can act as a platform on which stepwise assembly of H3–H4 tetramers can occur.

### The Vps75 tetramer is reconfigured upon H3–H4 binding

**Figure 5.**
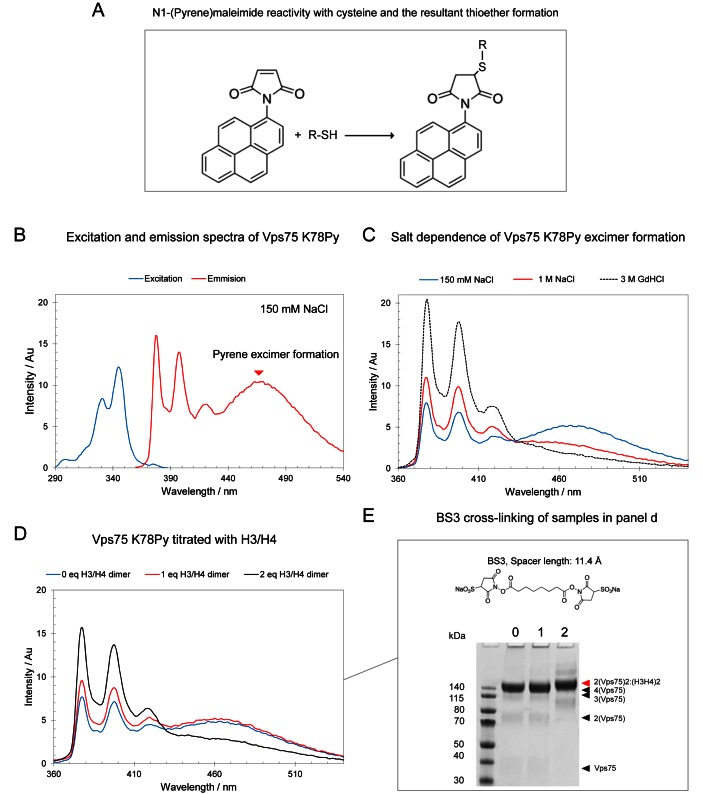
The Vps75 tetramer is remodeled upon histone binding. Pyrene excimer formation upon tetramerization of Vps75 K78C labeled with N1-(Pyrene)maleimide is quenched by histones H3–H4 but histones remain associated with the Vps75 tetramer suggesting thje Vps75 tetramer is remodeled upon histone binding. (**A**) Reaction scheme of N1-(Pyrene)maleimide reacting with a reduced cysteine side chain. (**B**) The excitation and emission spectra of Vps75 pyrene labeled at K78C forming the side chain K78Py ([(Vps75)_2_] = 8 μM, 0.36 nmol). Pyrene excimer formation at 470 nm is a result of two pyrene labels coming into close proximity across the Vps75 tetramerization interface at 150 mM sodium chloride. (**C**) The salt dependence of pyrene excimer formation ([(Vps75)_2_] = 4 μM, 0.18 nmoles) and total loss upon treatment with guanidine hydrochloride (GdHCl). (**D**) The addition of histones results in the loss of the Vps75 K78Py excimer at 400 mM sodium chloride ([(Vps75)_2_] = 4 μM). (**E**) SDS-PAGE analysis of samples (4-12% Bis–Tris NuPAGE gels) from part (D) cross-linked with Bis[sulfosuccinimidyl] suberate (BS3 2 μl, 50 mM), the number above each lane represents the number of equivalents of H3–H4 dimers added to 1 equivalent of Vps75 K78Py dimer.

The highly acidic concave surface between the two globular domains of Vps75 monomers is thought to be the histone binding surface (7). Within a Vps75 tetramer the cavity formed by this surface is too small to accommodate a H3–H4 tetramer (18) (Figure 1). This suggests that the structure adopted by the Vps75 tetramer in the absence of histones may require reconfiguration to allow histone binding. To probe the structure of the Vps75 tetramer upon histone binding, the fluorescent reagent N1-(pyrene)maleimide (pyrene – Figure 5A), was coupled to a cysteine residue introduced at lysine 78 (K78Py). This residue had previously been shown to come into close proximity with the corresponding lysine at the same position on the opposing dimer (18). When two pyrene fluorophores come into close proximity they display a characteristic ‘excimer’ peak at ∼470 nm in their emission spectrum (38). Upon labeling Vps75 K78C with pyrene (K78Py) the characteristic pyrene excimer was observed under conditions that favour the Vps75 tetramer (Figure 5B). This supports our previous observation that the K78 residues on opposing dimers come into close proximity across the tetramerization interface (18) demonstrating that the pyrene labels do not interfere with tetramerization of Vps75. The pyrene excimer was lost at high salt and under denaturing conditions (Figure 5C). As such the pyrene excimer was used as a readout of Vps75 tetramerization.

When histones H3–H4 were titrated into the pyrene labeled Vps75 tetramer, two equivalents of H3–H4 dimers to one equivalent of Vps75 dimer were sufficient to quench the pyrene excimer (Figure 5D). This indicates that the pyrene fluorophores have moved sufficiently far apart (>10 Å) to reduce excimer formation (39). In addition, cross-linking the titration points from the excimer experiment with the amine bifunctional cross-linker Bis[sulfosuccinimidyl] suberate (BS3) results in the generation of a high Mw species (red arrow, Figure 5E) similar in size to that previously assigned as a Vps75 tetramer bound to a H3–H4 tetramer (red arrows, Figure 4A–C). Note: the higher cross-linking efficiency of this product and the Vps75 tetramer compared to DTSSP (Figure 3B and E) is due to the higher molar ratio of BS3 used in this instance. This result suggests the Vps75 tetramer is re-configured rather than completely dissociated upon histone binding which quenches the pyrene eximer.

### A dimer of Vps75 can bind a dimer of H3–H4 in the presence of Asf1

The BS2G cross-linking titrations suggest that dimeric Vps75 binds to a H3–H4 dimer in a manner which does not compete with H3–H4 tetramerization (Figure 4) and explains previous observations that Vps75 binds a H3–H4 tetramer (15). This suggests that Vps75 interacts with the lateral surface of H3–H4 that maps the path of inner gyre of DNA in the nucleosome, but does not overlap with the H3–H4 tetramerization interface. As such Vps75 binding to H3–H4 may be compatible with Asf1, which binds to the H3–H4 tetramerization interface (19,20). To test this hypothesis we performed analytical gel filtration experiments.

**Figure 6.**
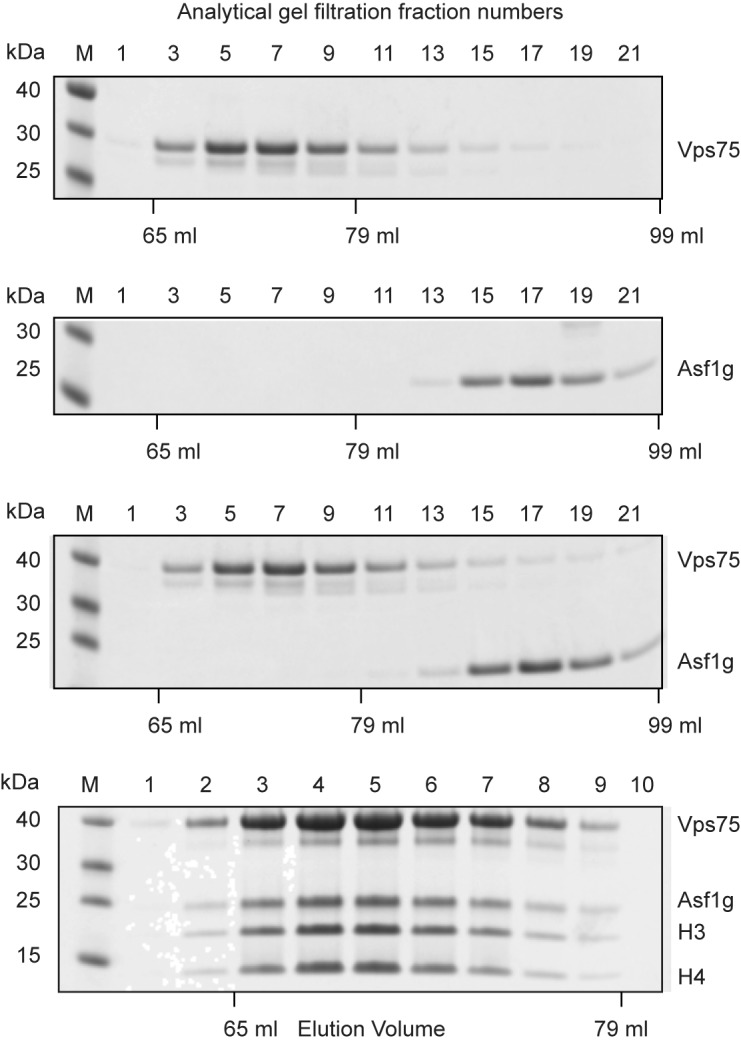
Vps75 binding to H3–H4 is compatible with Asf1 binding. SDS-PAGE analysis (4–12% Bis–Tris NuPAGE gels, Invitrogen) of fractions from the analytical gel filtration performed on a preparative scale using a HiLoad 16/600 Superdex 200 column (GE Healthcare). To span the peaks of Vps75 and Asf1, every other 2 ml fraction was analysed by SDS-PAGE as reflected by their fraction numbers (fractions 1–21). In contrast each fraction of the eluting Vps75–Asf1–H3H4 complex is shown (fractions 1–10). Vps75 does not interact with Asf1g in the absence of histones but can form a co-chaperone complex in the presence of histone H3–H4. M = PageRuler Prestained Protein Ladder (Thermo Scientific).

In the absence of histones, Vps75 does not co-elute with Asf1 from size exclusion chromatography (Figure 6). When a dimer of H3–H4 is added to a mixture of equimolar amounts of Vps75 dimer and Asf1, all four proteins co-fractionate, with a shift in the elution profile peak consistent with the formation of a higher order complex (Figure 6 and Supplementary Figure S5). Cross-linking analysis of Vps75 with Asf1 and H3–H4 confirmed the 2:1:1 stoichiometry of the duel histone chaperone complex, which migrates during SDS-PAGE at the same molecular weight as a Vps75 dimer to a H3–H4 tetramer (blue arrow and red star respectively, Supplementary Figure S4). In a similar manner, Nap1 was observed to bind H3–H4 in complex with Asf1 (Supplementary Figure S5). This suggests that both Vps75 and Nap1 share a similar mode of interaction with H3–H4 that does not overlap with the binding surface of Asf1.

### Probing the mode of interaction of Vps75 with Asf1-H3–H4 through a combination of spin labeling PELDOR and cross-linking derived distance restraints

**Figure 7.**
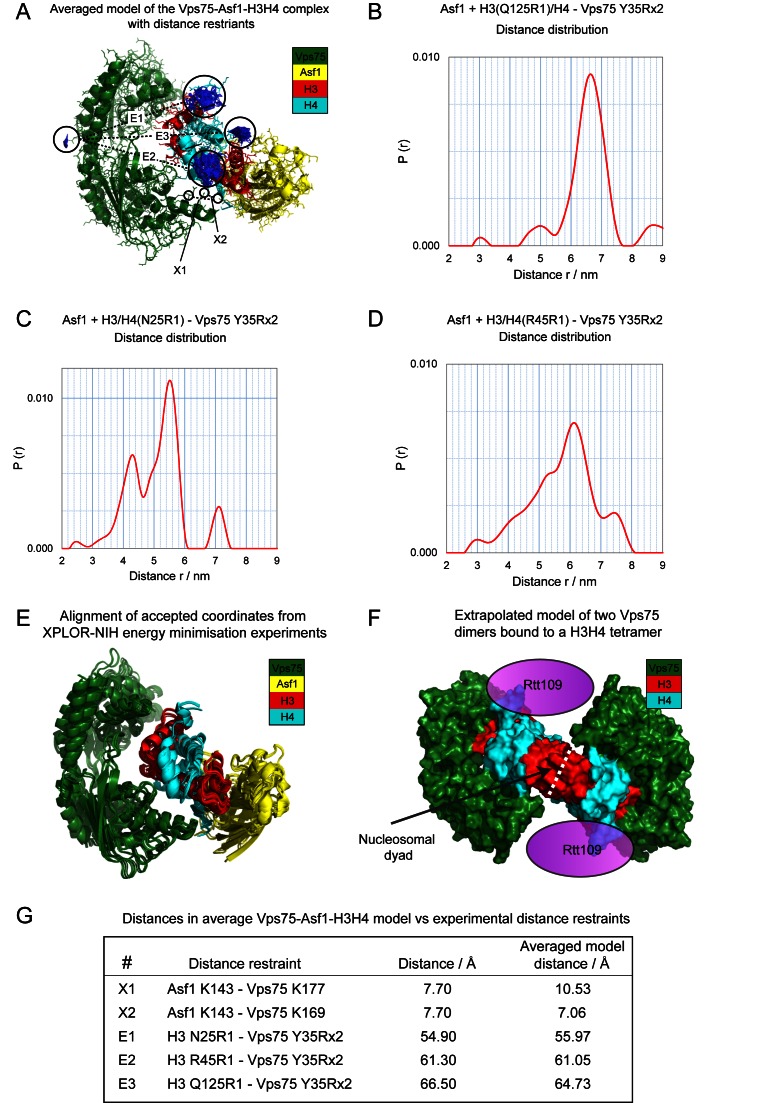
The mode of interaction of Vps75 with Asf1-H3H4. (**A**) The average model of the Vps75–Asf1–H3–H4 complex derived from rigid body energy minimization experiments used to dock together the crystal structures of Vps75 (PDB code 2ZD7 (9) with the associated spin label ensemble Y35Rx2) and Asf1–H3–H4 (PDB code 2HUE(19) with associated H3 Q125R1, H4 N25R1 and H4 R45R1 spin label ensembles) against PELDOR and cross-linking distance restraints in XPLOR-NIH. Proteins chains for Vps75 (green), Asf1 (yellow), H3 (red) and H4 (cyan) in cartoon and stick representation are depicted with associated spin label ensembles (blue). PELDOR distance restraints (E1–E3) and cross-linking distance restraints (X1 and X2) characterised by mass spectrometry (Supplementary Figure S7) are indicated and identified in (G). PELDOR derived distance distributions between spin labels positioned at (**B**) H3 Q125R1 and Vps75 Y35Rx2, (**C**) H4 N25R1 and Vps75 Y35Rx2 and (**D**) H4 R45R1 and Vps75 at Y35Rx2 produced as a result of Tikhonov regularization of background corrected PELDOR data (Supplementary Figure S6) in DeerAnalysis2013 (60). (**E**) Alignment of accepted coordinates from rigid body energy minimization experiments in XPLOR-NIH used to dock together the crystal structures of Vps75 (with the associated spin label ensemble Y35Rx2) and Asf1-H3–H4 (with associated H3 Q125R1, H4 N25R1 and H4 R45R1 spin label ensembles). Energy minimization experiments were initiated from 500 randomly oriented Vps75 and Asf–H3–H4 starting coordinates (not shown). Solutions were ranked by NOE energy term. Cut-off levels of *E*(NOE) <170 kcal mol^−1^ and *E*(total) < 6900 kcal mol^−1^ were chosen to isolate models that best fit the experimental distance restraints. The 62 energy minimized coordinates that met these criteria were aligned by backbone atoms (E) and averaged (A). (**F**) The model extrapolated by aligning a Vps75–Asf1-H3–H4 complex to each dimer of H3–H4 in the H3–H4 tetramer (extracted from the crystal structure of the histone octamer—PDB code 1TZY (54)) and removing the two copies of Asf1. The mode of interaction of Vps75 with a H3–H4 dimer in this complex is compatible with a H3–H4 tetramer in the absence of Asf1 and the H3–H4 tetramer can accommodate two Vps75 dimers. (**G**) The deviations between experimental distance restraints and measured distances in the average model.

The occlusion of the histone H3–H4 tetramerization interface by Asf1 (19,20), reduces the potential for higher order oligomers and makes the Vps75–Asf1–H3–H4 (VAH) complex particularly tractable for structural studies. As crystal structures of both Asf1–H3–H4 (19,20) and dimeric Vps75 (9) have been reported previously, we designed experiments to obtain distance restraints in order to dock the two structures together. Firstly, we designed a spin-labeling strategy to obtain distance information from the VAH complex via pulsed electron-electron double resonance (PELDOR) experiments. To simplify distance information, we designed spin-labeling sites that would lead to a two spin-system in the VAH complex. To achieve this, Vps75 was cross-link spin labeled with 3,4-bis(MTSL) via a cysteine mutation at tyrosine 35, creating a singly spin labeled Vps75 dimer (Vps75 Y35Rx2), as reported previously (18). Distances were then measured from Vps75 Y35Rx2 to histone labeling sites at either H3 Q125R1, H4 N25R1 or H4 R45R1 (Figure 7A). For example, H3 Q125R1 represents glutamate 125 of histone H3 that has been mutated to cysteine and spin labeled with MTSL forming the spin labeled side chain R1. Samples also contained 1.5 equivalents of globular, cysteine-free Asf1 (Asf1g residues 1–164) to each equivalent of Vps75 dimer and H3–H4 dimer. The excess Asf1 excludes any contribution from tetrameric H3–H4 in the sample and ensured only distance information between Vps75 and histones was extracted.

In PELDOR experiments, the dipole–dipole interaction between two spin labeled sites is inversely proportional to the cube of the distance and is manifested as a modulation in the dipolar time evolution data (40). Distance distributions can therefore be extracted from PELDOR data, reporting on the structure of the underlying protein or protein complex. Oscillations were observed in the raw dipolar evolution functions of Vps75 Y35Rx2—H3 Q125R1, Vps75 Y35Rx2—H4 N25R1 and Vps75 Y35Rx2—H4 R45R1 consistent with the Vps75 Asf1 H3–H4 complex being structurally well-defined (Supplementary Figure S6). Upon Tikhonov regularization of the background-corrected dipolar evolution function, distance distributions with modal distances of 6.65, 5.49 and 6.13 nm were obtained for Vps75 Y35Rx2 to H3 Q125R1 (Figure 7B), H4 N25R1 (Figure 7C) and H4 R45R1 (Figure 7D), respectively.

To obtain additional structural constraints, we adopted a cross-linking approach. This involved cross-linking the purified Vps75 Asf1g H3–H4 complex with 20 equivalents of isotope labeled BS2G-d0/d4. After cross-linking, the complex was re-purified and fractions spanning the elution peak were analysed by SDS-PAGE. This shows a ladder of partially cross-linked species with the upper most species migrating slightly above the 115 kDa marker (Supplementary Figure S7A) consistent with previous cross-linking analysis of the Vps75 Asf1g H3–H4 complex (Supplementary Figure S4). These complexes were subjected to trypsin digestion to release cross-linked peptides for MS/MS analysis. MS/MS data were searched for BS2G-d0/d4 cross-links using Hekate (34). 21 cross-links were identified that passed stringent selection criteria (Supplementary Table S2). Of these, the cross-links between Asf1 K143 and Vps75 at either K177, or K169 (Supplementary Figure S7B and SC), were most useful as distance constraints, because they are resolved in Vps75 and Asf1–H3–H4 crystal structures.

### Defining a structural model for the interaction of Vps75 with Asf1–H3–H4 using rigid body energy minimizations against distance restraints in XPLOR-NIH

Crystal structures of the Vps75 dimer (PDB code: 2ZD7) and Asf1 bound to histones (PDB code 2HUE) with added spin label ensembles were docked together by energy minimization experiments in XPLOR-NIH, using a combination of the 3× PELDOR and 2× cross-linking distance restraints described above. Following minimization of 500 starting coordinates in which the relative orientations of Vps75 and Asf1–H3–H4 were randomized (not shown), a total of 62 solutions were accepted based on NOE energy terms and aligned via backbone atoms (Figure 7E) to their average calculated structure (Figure 7A). Following alignment, accepted coordinates had an average RMSD of 4.68 Å for backbone atoms, and 5.05 Å for all non-hydrogen atoms, to the mean structure (Figure 7A). All experimental distance restraints were satisfied within 3 Å in the final averaged model of the Vps75–Asf1–H3–H4 complex (Figure 7G).

Each monomer of Vps75 contains two distinct binding sites (sites A and B), which contact different regions of the H3–H4 dimer in a mutually exclusive manner. The asymmetry of the H3–H4 dimer is accommodated by the symmetrical dimer of Vps75 using site A of one Vps75 monomer and site B of the opposing Vps75 monomer to simultaneously bind a H3–H4 dimer. The binding of one H3–H4 dimer (with Asf1) partially obscures the binding site required for a second H3–H4, thus providing a rationale for why a Vps75 dimer only binds one H3–H4 dimer when there are two A and B sites within the dimer. In addition, cross-linking analysis of the Vps75 Asf1 H3–H4 complex shows that the 2:1:1 complex is the more favourable stoichiometry (Supplementary Figure S4) and oscillation depths recorded in the PELDOR experiments are indicative of two spins interacting, one on the Vps75 dimer and one on the H3–H4 dimer in the presence of Asf1 (Supplementary Figure S6).

The mode of interaction of Vps75 with Asf1–H3–H4 is compatible with conclusions made from biochemical observations described here and previously (15,18). Firstly, the mode of interaction of a Vps75 dimer with the H3–H4 dimer would be compatible with H3–H4 tetramerization in the absence of Asf1 and tetrameric H3–H4 could accommodate two Vps75 dimers. These complexes were observed in cross-linking titrations of Vps75 with histones (Figure 4 and Supplementary Figure S3) and Vps75 was previously shown to bind canonical H3–H4 tetramers (18). Secondly, extrapolation of the model of the VAH complex to a model of two Vps75 dimers bound to one H3–H4 tetramer, shows that neither direct contacts nor steric clashes, are made between Vps75 dimers (Figure 7F). This supports the pyrene cross-linking studies that suggest the Vps75 tetramer is reconfigured upon histone H3–H4 binding without the loss of a Vps75 dimer from the complex (Figure 5). The model also suggests that conserved residues within Vps75 contribute to the interface with H3–H4 (Supplementary Table S3 and Figure S2D). Finally, a PELDOR distance measurement between Vps75 Y35Rx2 and H3 G132Rx2 (sites used to singly spin label Vps75 dimers and H3–H4 tetramers, respectively), was in close agreement with the structural model of the VAH complex (Supplementary Figure S8). We conclude that Vps75 binds both tetramers and dimers of H3–H4 using the same interaction surface.

## DISCUSSION

In this study, we present a crystal structure of Vps75 in which two related forms of a Vps75 tetramer are observed (Figure 1). Most notably the symmetrical form of the Vps75 tetramer is in close agreement with the conformation observed in solution (18) (Supplementary Figure S1). Furthermore, H3H4 binding maps to surfaces of Vps75 (Figure 7) that are sequestered upon tetramerization (Figure 1) (18). This supports the idea that tetrameric Vps75 may fulfil a self-chaperoning function in the absence of histone cargo. The tetrameric form of Vps75 may be especially important outside of S-phase owing to the fact that Vps75 expression is constitutive (41), whereas the expression of its binding partner, the acetyltransferase Rtt109, is restricted to S-phase (22).

Vps75 binds histones H3 and H4 in the presence of Asf1 to form a well-defined complex (Figure 6). Using a combination of PELDOR measurements and cross-linking distance restraints a structural model of the VAH complex was generated (Figure 7). The model shows that whilst cross-links between Vps75 and Asf1 were observed, these distance restraints can be satisfied without substantial contacts between Vps75 and Asf1 (Figure 7A). Thus, it is the association of Vps75 with H3–H4 that allows Asf1 to co-fractionate with Vps75–H3–H4 (Figure 6 and Supplementary Figure S5). We also observe that the related chaperone, Nap1, is capable of binding histones H3–H4 in concert with Asf1 (Supplementary Figure S5). These findings parallel the ability of Asf1 to form co-chaperone complexes with NASP, MCM2, RbAp46, HIRA (42–48). This is an emerging theme in histone dynamics and suggests that the engagement of Asf1 with the H3–H4 tetramerization interface does not prevent the association of H3–H4 with a significant subset of histone chaperones.

Our observation that Vps75 can form a ternary complex with histone H3–H4 dimers and Asf1 provides potential insight into the interplay between these chaperones during Rtt109 catalysed acetylation events (4,24,49–51). In *S. cerevisiae*, H3 K9ac is catalysed by both Rtt109 and Gcn5 whereas H3 K56ac is solely dependent on the presence of Rtt109 (24). Loss of H3 K9ac in both asf1Δ/gcn5Δ and vps75Δ/gcn5Δ double mutant backgrounds shows that both Asf1 and Vps75 are involved in promoting Rtt109 directed H3 K9 acetylation (24). Further support for the delivery of H3–H4 to Vps75-Rtt109 by Asf1 stems from the observation that Vps75 and Rtt109 can interact with Asf1 H3–H4 *in vitro* and in yeast extracts (52). The VAH complex described here potentially provides a structural basis for the previous observations indicating that both Vps75 and Asf1 are required for Rtt109 mediated H3 K9ac.

In contrast to H3 K9ac, H3 K56ac directed by Rtt109 is dependent on Asf1 but not Vps75 (21,51). Given the ability of Asf1 to form co-complexes with multiple chaperones it seems possible that another chaperone may substitute for Vps75 in H3 K56ac but lack the ability to direct Rtt109 activity towards H3 K9. The positioning of different histone residues with respect to the Rtt109 active site is likely to play an important role in determining specificity within these chaperone complexes. Residues in the H3 N-terminal tail were observed to cross-link to multiple sites in the VAH complex. This suggests that the H3 tails remains somewhat dynamic. Nonetheless, the majority of H3 tail cross-links map to the interface of Vps75–H3H4 closest to Asf1 (Supplementary Table S2). These include: Vps75 K78 (H3 K9, K14, K18), Vps75 K177 (H3 K18, K27), Asf1 K143 (H3 K18) and H4 K91 (H3 K14, K18, K27). Dynamics in the H3 tail may support the ability of Vps75-Rtt109 to acetylate multiple sites in the H3 N-terminal tail (53). Cross-links between H3 K56 (K18, K64) suggest the H3 αN helix may occupy a distinct conformation not seen in the histone octamer (the H3 K56-K64 Nϵ-Nϵ distance in the octamer is ∼21 Å with poor geometry for cross-linking) (54). Conformational heterogeneity in this region of H3–H4 has been reported previously (33,55,56). In such an altered configuration it is possible that H3 K56 is less accessible for Rtt109 mediated acetylation (53).

Within the VAH complex it is possible to substitute Asf1 with a second H3–H4 dimer (Figure 7F). This in turn provides a binding site for a second Vps75 dimer in a histone sandwich-like configuration (Figure 7F). This provides a satisfying explanation for the loss of pyrene excimer (Figure 5) in a reconfigured tetrameric state and is also consistent with the multiple distinct assemblies observed during cross-linking titrations (Figure 4). In this configuration there is potential to accommodate one Rtt109 monomer per Vps75 dimer (Figure 7F). This could allow the H3 tail to be acetylated in trans providing a possible explanation for the preferential acetylation of H3–H4 tetramers by Vps75–Rtt109 (15). In this respect, it will be interesting to see if Rtt109 can simultaneously associate with Vps75, Asf1 and H3–H4 or whether there is a hand-over of H3–H4 between Vps75 and Asf1 during Rtt109 catalysis (and if so in which direction it occurs).

The ability of Vps75 dimers to bind H3–H4 dimers in a manner compatible with H3–H4 tetramerization provides Vps75 with the capacity to act either as a platform to assemble H3–H4 tetramers from cognate histone fold dimers, or to catch H3–H4 tetramers evicted from chromatin. Consistent with this, Vps75 does not shield the entire DNA binding surface of the H3–H4 tetramer (Figure 7F). The accessibility of the strong DNA binding sites around the dyad axis of symmetry provides a means by which chaperone associated H3–H4 tetramers could be directly deposited onto DNA (15). While the structural studies reported here focus on Vps75, it seems likely that Nap1 interacts with histones H3 and H4 in a similar mode. In addition to sequence and structural homology, both proteins form homo-tetramers (18) and associate with Asf1 bound H3–H4 dimers (Supplementary Figure S5). As a result it is possible that other Nap1-fold histone chaperones engage with histones H3 and H4 in a similar way to Vps75 (Figure 7). Current evidence supports the notion of chromatin evicted H3–H4 being recycled as tetramers during both DNA replication-independent and replication-dependent deposition (57,58). The ability to engage with tetrameric H3–H4 provides Nap1-fold chaperones with the potential to act to maintain the integrity of H3–H4 tetramers that have been evicted from chromatin. As both Nap1 and Vps75 are linked to transcription coupled processes including the suppression of cryptic transcription (59), they are well placed to perform this function during replication independent histone turnover in addition to their distinct functions during S-phase.

## Supplementary Material

SUPPLEMENTARY DATA
