# Supplementary material for: The histone chaperone Vps75 forms multiple oligomeric assemblies capable of mediating exchange between histone H3–H4 tetramers and Asf1–H3–H4 complexes
Source: Nucleic Acids Res. 2016 Apr 1;44(13):6157–72. doi: 10.1093/nar/gkw209 (PMC5291247; doi:10.1093/nar/gkw209)
Supplement: SUPPLEMENTARY DATA [file supp_44_13_6157__index.html]

The histone chaperone Vps75 forms multiple oligomeric assemblies capable of mediating exchange between histone H3–H4 tetramers and Asf1–H3–H4 complexes — SUPPLEMENTARY DATA 

# The histone chaperone Vps75 forms multiple oligomeric assemblies capable of mediating exchange between histone H3–H4 tetramers and Asf1–H3–H4 complexes

## SUPPLEMENTARY DATA

- SUPPLEMENTARY DATA
- SUPPLEMENTARY DATA
