# Supplementary material for: The histone chaperone Vps75 forms multiple oligomeric assemblies capable of mediating exchange between histone H3–H4 tetramers and Asf1–H3–H4 complexes
Source: Nucleic Acids Res. 2016 Apr 1;44(13):6157–72. doi: 10.1093/nar/gkw209 (PMC5291247; doi:10.1093/nar/gkw209)
Supplement: SUPPLEMENTARY DATA [file supp_gkw209_nar-03698-m-2015-File010.pdf]

Supplementary DATA

- "Asymmetric_Vps75_tetramer.pdb" Coordinates of asymmetric form of Vps75 from crystal lattice.
- "Symmetric_Vps75_tetramer.pdb" Coordinates of symmetric form of Vps75 from crystal lattice.
- "VAH_aligned.pdb" Accepted coordinates from XPLOR-NIH rigid body energy minimization with energy terms visible if opened in a text editor.
- "VAH_average.pdb" Average of accepted coordinates with RMSD visible if opened in a text editor.
- "Consurf_asymm-tetramer.pse" ConSurf amino acid conservation of Vps75 in the context the asymmetric tetramer.
- "Consurf_symm-tetramer.pse" ConSurf amino acid conservation of Vps75 in the context the asymmetric tetramer.
- "Consurf_VAH.pse" ConSurf amino acid conservation of Vps75 in the context the Vps75-Asf1-H3/H4 average structure.
- "B49GSVTT211-alignment.aln" Clustal W V 1.83 multiple sequence alignment of 100 target sequences identified by blastp using Vps75 protein sequence (YNL246W) against Fungi (taxid:4751) protein sequences.

Supplementary MATERIALS AND METHODS

Structural analysis of conserved residues of Vps75

Amino acid conservation analysis of Vps75 was performed by uploading the relevant PDB file of a Vps75 tetramer to ConSurf webserver (1-4) with a multiple sequence alignment and query sequence of *Saccharomyces cerevisiae* Vps75 (gi|6324083). Multiple sequence alignments were generated in Clustal W v1.83 format using the NCBI blasp algorithm and the protein sequence of Vps75 (YNL246W) against the Reference proteins database (refseq_protein) in Fungi (taxid:4751) with a maximum of 100 target sequences and otherwise default settings (Supplementary file "B49GSVTT211-alignment.aln"). The ConSurf score for each residue in Vps75 generated was applied to the structures of the Vps75 tetramer and Vps75-Asf1-H3H4 complex (Figure S9). Tetramerisation interface residues were identified using the PDBePISA webserver (5) and cross-referenced to ConSurf scores (Table S3). Pymol session files are provided in the supplementary data for further analysis of amino acid conservation of Vps75 in the context of the asymmetric Vps75 tetramer (Supplementary file "5_Consurf_asymmetric_tetramer.pse"), the symmetric Vps75 tetramer (Supplementary file "6_Consurf_symmetric_tetramer.pse") and the Vps75-Asf1-H3H4 complex (Supplementary file "7_Consurf_VAH.pse").

Supplementary FIGURE LEGENDS

Figure S1. A comparison of the in solution structure of Vps75 and the symmetrical form of Vps75 crystallised. An alignment of the model of the Vps75 tetramer derived from PELDOR distances measurements (6) to the crystal structure of the symmetrical Vps75 tetramer (PDB code 5AGC). The overall arrangement of the two Vps75 dimers in the published model (6) of the Vps75 tetramer derived from PELDOR distance measurements is within close agreement to symmetrical crystallised form of the Vps75 tetramer.

Figure S2. Structural analysis of the amino acid conservation of Vps75. Residues of Vps75 coloured by ConSurf score in the context of (A) the Vps75 dimer - with cartoon representations for orientation in greens, (B) the asymmetrical form of the Vps75 tetramer, (C) the symmetrical form of the Vps75 tetramer and (D) the model of the Vps75-Asf1-H3H4 complex. Components of complexes not coloured for conservation are depicted in orange ribbon. This includes a Vps75 dimer in (A-C) and Asf1-H3/H4 in (D).

Figure S3. SEC-MALS analysis of Vps75-H3H4. (A) SEC-MALS analysis of Vps75-H3H4, mixed in a 1:1 dimer ratio, eluted from Superdex 200 10/300 GL column (GE Healthcare) at 150 mM NaCl, 5 mM $MgCl_2$, 20 mM HEPES-KOH and 5 mM β-mercaptoethanol. No free histone peak was observed in the Vps75 H3/H4 chromatogram despite evidence of dissociation of the complex. Chromatogram is normalised by the elution peak. (B) SDS-PAGE analysis (4-12% Bis –Tris NuPAGE gels) of fractions from the SEC-MALS analysis of cross-linked Vps75. Blue arrows highlight H3 and H4 bands co-eluting with Vps75.

Figure S4. The stoichiometry of the Vps75 Asf1 H3H4 complex using amine reactive cross-linking. SDS-PAGE analysis of Vps75 incubated with increasing concentrations of H3H4 in the presence or absence of globular Asf1 (1-164) and cross-linked with BS_2G as in part (A). Colour coded arrows and stars next to major cross-linked species match the cross-linked species annotation and are additionally consistent with the relevant species in Figure 4.

Figure S5. Nap1 and Vps75 bind histones H3-H4 in the presence of Asf1g. Analytical gel filtration shows that Nap1 and Vps75 can form a complex with H3-H4 and the globular domain of Asf1 (Asf1g - residues 1-164). Thus Nap1 likely interacts with H3-H4 in a similar manner to Vps75. SDS-PAGE analysis of fractions from analytical gel filtration runs on a Superdex 200 PC 3.2/30 column (GE Healthcare) fraction numbers and elution volumes indicated, 20 µl of 80 µl fractions were run on 4-12% Bis –Tris SDS-PAGE gels (Invitrogen). Equimolar amounts of Nap1 dimer, Asf1g, H3-H4 tetramer, were run separately and Nap1+Asf1+H3-H4 run in a 2:1:1:1 stoichiometry. Vps75 analytical gel filtration analyses were performed using the same stoichiometries. Asf1-H3-H4 co-elutes with Nap1 and Vps75 with a shift in elution volume of the two complexes, due to the higher molecular weight of Nap1 vs Vps75, confirming the formation of the respective Nap1-Asf1-H3-H4 (NAH) and Vps75-Asf1-H3-H4 (VAH) complex.

Figure S6. Raw and background corrected dipolar evolution functions for PELDOR measurements. PELDOR data showing the dipolar evolution functions with background fits (left) and background corrected dipolar evolution functions (right) for the Vps75-H3/H4-Asf1 complex cross-link spin labelled at Vps75 Y35Rx2 and spin labelled at either (A) H3 Q125R1, (B) H4 N25R1 or (C) H4 R45R1. Background corrected dipolar evolutions functions were used to calculate distance distributions via Tikhonov regularisation in DeerAnalysis 2013(7) (Figure 7B-D).

Figure S7. Cross-linked MS/MS analysis of the Vps75-Asf1-H3H4 complex. (A) SDS-PAGE analysis, on a 4-12% Bis –Tris SDS-PAGE gels (Invitrogen), of fractions of Vps75g (residues 1-225), Asf1g (residues 1-164) and H3H4 cross-linked minimally with BS_2G and separated using a Superdex 200 10/300 GL (GE Healthcare) column. Un-crosslinked Vps75g (cleaved tag), Asf1g (cleaved tag), H3 and H4 are indicated by green, yellow, red and cyan arrows respectively. The upper most cross-linked band indicated by the black arrow, migrates consistent with the 2Vps75g+H3H4+Asf1g complex as seen in Figure S3B. Below, shows MS2 spectra details output by Hekate(8) for (B) the Vps75 K177 – Asf1 K143 cross-linked peptide assignment and (C) the Vps75 K169 – Asf1 K143 cross-linked peptide assignment.

Figure S8. The distance between a singly spin labelled Vps75 dimer and a singly spin labelled H3H4 tetramer matches the expected distance from the VAH complex model. (A) The location of spin labelling sites H3 G132Rx2 used to singly label the H3H4 tetramer - model derived from the crystal structure of the nucleosome with residues 1:42 of H3 and 1:19 of H4 omitted for clarity (PDB code 1KX5)(9), and the location of the Vps75 labelling site Y35Rx2 on the respective crystal structure (PDB code 2ZD7)(10). Rx2 labelling sites were simulated in XPLOR-NIH as reported (11). SDS-PAGE analysis (4-12% Bis –Tris NuPAGE gels) of sample submitted for PELDOR analysis with both cross-linked spin labelled Vps75 and H3 indicated. (B) A model of the mode of interaction of the H3H4 tetramer with a dimer of Vps75, extrapolated from the Vps75-Asf1-H3H4 model, with spin label ensembles for H3 G132Rx2 and Vps75 Y35Rx2 in blue. (C) PELDOR data for the Vps75-H3/H4 complex between Vps75 Y35Rx2 and H3 G132Rx2 labelling sites. The experimental distance distribution in red (modal distance 6.04 nm) is overlaid with an MTSSLwizard (12) calculated distance distribution in blue (average distance 6.16 nm) between spin label ensembles depicted in (B) normalised to the experimental P(r) maximum.

Table S1. Parameters used for pyrene fluorescence studies on the Cary Eclipse Fluorescence Spectrophotometer.

Table S2. Peptides identified from MS/MS analysis of the Vps75/Asf1/H3H4 complex. Complexes isolated as described in S7A were subject to trypsin digestion to release cross-linked peptides for MS/MS analysis. Data from MS/MS analysis was searched for BS2G-d0/d4 cross-links using Hekate (8) with an additional decoy search enabling false discovery rate determination for each cross-linked species. Only cross-linked peptides ≥ 5 residues on A and B chains of the cross-linked

peptide, a false discovery rate of ≤ 5% and were forwarded for further consideration. In addition ≥2 BS2G-d0/d4 isotope pairs were observed for all accepted cross-links. The most abundant cross-link, observed 36 times, was between Vps75 K163 and Vps75 K189 which are ~ 14 Å apart (Nε-Nε distance) in the crystal structure of Vps75 (PDB code 2ZD7) (10) – a distance easily bridged by the BS2G cross-linking radius (7.7 Å) and side chain dynamics. Other notable intra-protein cross-links include H3 K18-K27, H3 K18-K56 and H3 K56-K64, these intra-H3 cross-links suggest significant dynamics in the H3 tail and αN helix which together constitute further evidence of conformational dynamics of this region as reported previously (13-15). Although these cross-links provide confidence in the assignment of cross-linked peptides by Hekate (8) they do not provide useful restraints for docking purposes. In contrast cross-links between Asf1 K143 and Vps75 at K169 or K177 were observed (Figure S7BC) and, as these residues are resolved in Vps75 and Asf1-H3/H4 crystal structures, could be used as distance restraints. Although Asf1 and Vps75 were not observed to co-fractionate in the absence of histones (Figure 6) these cross-links suggest regions of close proximity between the chaperones in the VAH complex.

Table S3. ConSurf scores for amino acid conservation of residues in Vps75 involved in the tetramerisation interfaces and the interface between Vps75 and H3/H4 in the Vps75-Asf1-H3/H4.

1. Ashkenazy, H., Erez, E., Martz, E. and Pupko, T. (2010) ConSurf 2010: calculating evolutionary conservation in sequence and structure of proteins and nucleic acids. *Nucleic acids …*.
2. Celniker, G., Nimrod, G. and Ashkenazy, H. (2013) ConSurf: using evolutionary data to raise testable hypotheses about protein function. *Israel Journal of …*.
3. Landau, M., Mayrose, I. and Rosenberg, Y. (2005) ConSurf 2005: the projection of evolutionary conservation scores of residues on protein structures. *Nucleic acids …*.
4. Glaser, F., Pupko, T., Paz, I. and Bell, R.E. (2003) ConSurf: identification of functional regions in proteins by surface-mapping of phylogenetic information. ….
5. Krissinel, E. and Henrick, K. (2007) Inference of macromolecular assemblies from crystalline state. *Journal of molecular biology*, **372**, 774-797.
6. Bowman, A., Hammond, C.M., Stirling, A., Ward, R., Shang, W., El-Mkami, H., Robinson, D.A., Svergun, D.I., Norman, D.G. and Owen-Hughes, T. (2014) The histone chaperones Vps75 and Nap1 form ring-like, tetrameric structures in solution. *Nucleic acids research*, **42**, 6038-6051.
7. Jeschke, G., Chechik, V., Ionita, P. and Godt, A. (2006) DeerAnalysis2006—a comprehensive software package for analyzing pulsed ELDOR data. *Applied Magnetic …*.

8. Holding, A., Lamers, M., Stephens, E. and Skehel, J. (2013) Hekate: software suite for the mass spectrometric analysis and three-dimensional visualization of cross-linked protein samples. *Journal of proteome research*, **12**, 5923-5933.
9. Davey, C.A., Sargent, D.F., Luger, K., Maeder, A.W. and Richmond, T.J. (2002) Solvent mediated interactions in the structure of the nucleosome core particle at 1.9 a resolution. *Journal of molecular biology*, **319**, 1097-1113.
10. Park, Y.-J., Sudhoff, K., Andrews, A., Stargell, L. and Luger, K. (2008) Histone chaperone specificity in Rtt109 activation. *Nature structural & molecular biology*, **15**, 957-964.
11. Stevens, M.A., McKay, J.E., Robinson, J.L.S., Mkami, H.E.L., Smith, G.M. and Norman, D.G. (2016) The use of the Rx spin label in orientation measurement on proteins, by EPR. *Physical Chemistry Chemical Physics*.
12. Hagelueken, G., Ward, R., Naismith, J.H. and Schiemann, O. (2012) MtsslWizard: In Silico Spin-Labeling and Generation of Distance Distributions in PyMOL. *Applied Magnetic Resonance*, **42**, 377391.
13. Bowman, A., Ward, R., El-Mkami, H., Owen-Hughes, T. and Norman, D.G. (2010) Probing the (H3-H4)2 histone tetramer structure using pulsed EPR spectroscopy combined with site-directed spin labelling. *Nucleic acids research*, **38**, 695-707.
14. Elsässer, S.J., Huang, H., Lewis, P.W., Chin, J.W., Allis, C.D. and Patel, D.J. (2012) DAXX envelops a histone H3.3-H4 dimer for H3.3-specific recognition. *Nature*, **491**, 560-565.
15. Liu, C.-P.P., Xiong, C., Wang, M., Yu, Z., Yang, N., Chen, P., Zhang, Z., Li, G. and Xu, R.-M.M. (2012) Structure of the variant histone H3.3-H4 heterodimer in complex with its chaperone DAXX. *Nature structural & molecular biology*, **19**, 1287-1292.

Hammond CM et al. 2016
Figure S1

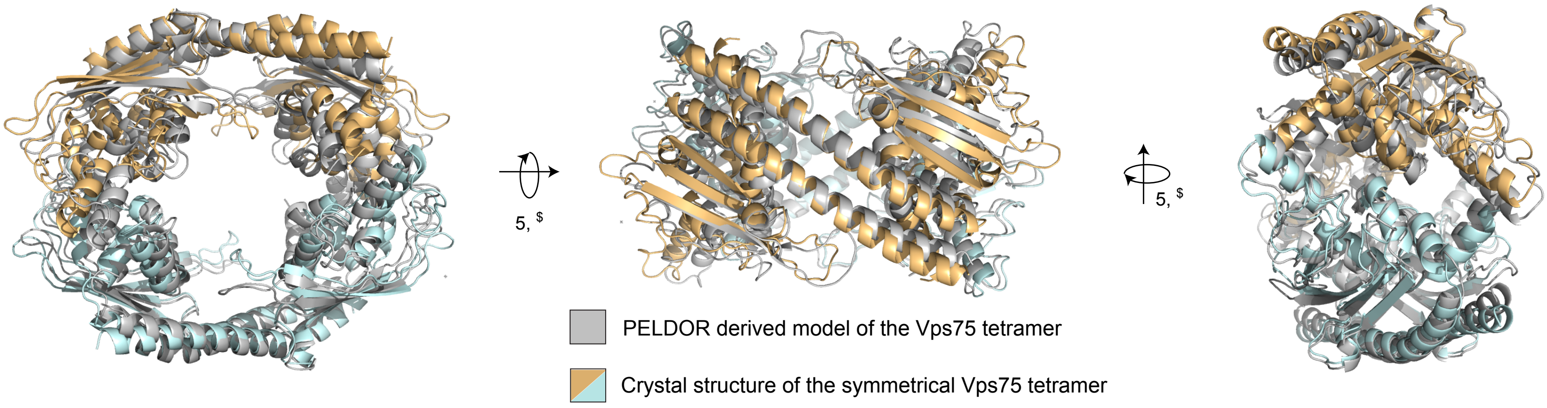

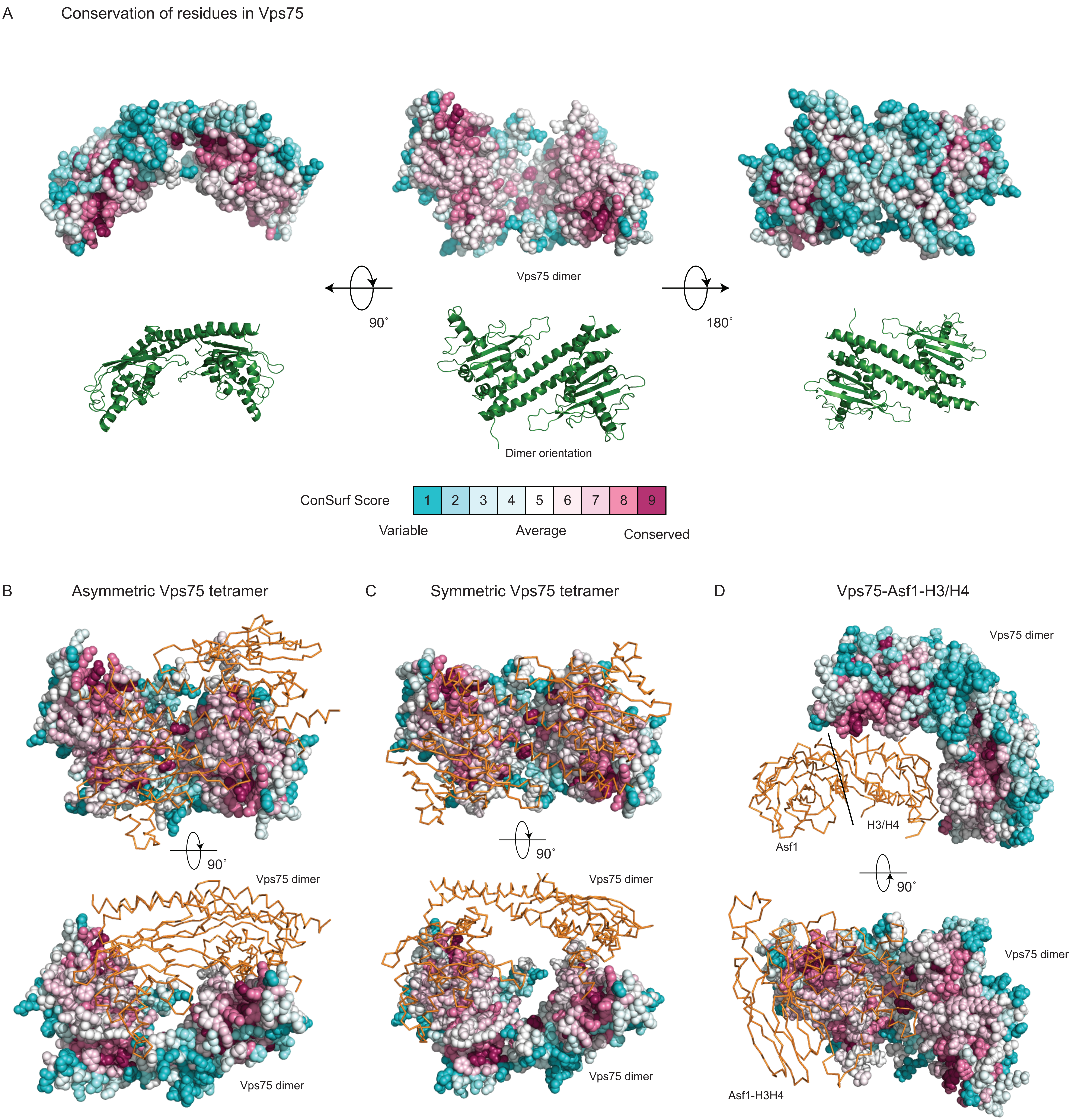
A
Conservation of residues in Vps75
Vps75 dimer
90˚
180˚
Dimer orientation
ConSurf Score
1
2
3
4
5
6
7
8
9
Variable
Average
Conserved
B
Asymmetric Vps75 tetramer
90˚
Vps75 dimer
Vps75 dimer
C
Symmetric Vps75 tetramer
90˚
Vps75 dimer
Vps75 dimer
D
Vps75-Asf1-H3/H4
Vps75 dimer
H3/H4
Asf1
90˚
Vps75 dimer
Asf1-H3H4

Hammond CM et al 2016
Figure S3

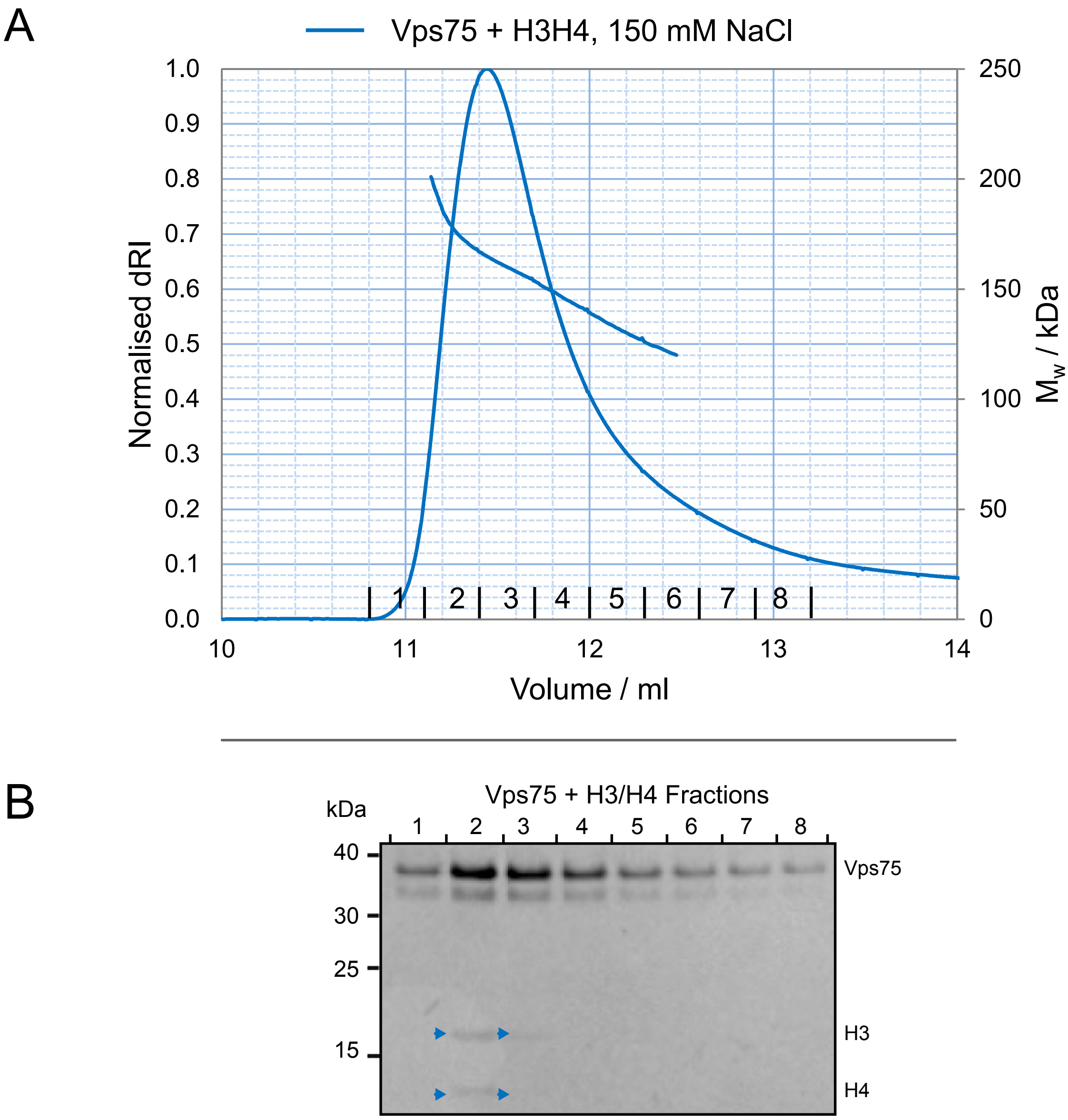

BS2G cross-linking of Vps75 with H3H4 ± Asf1

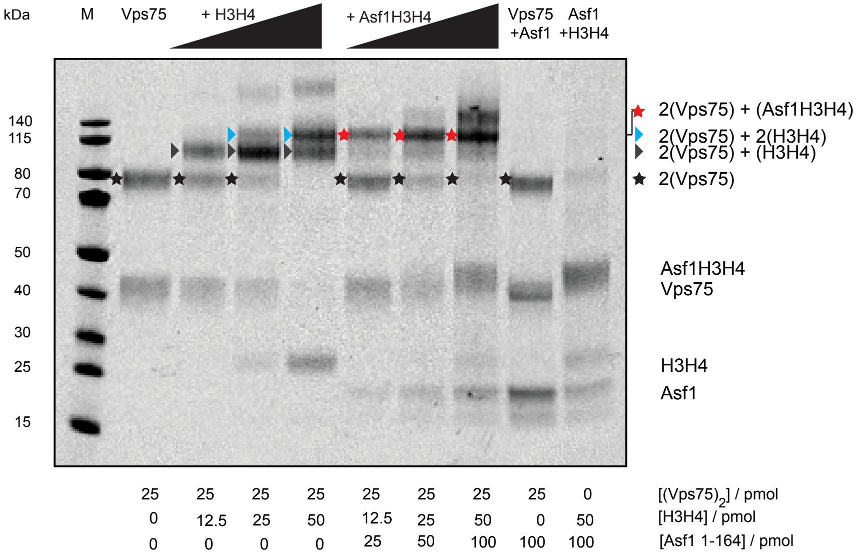

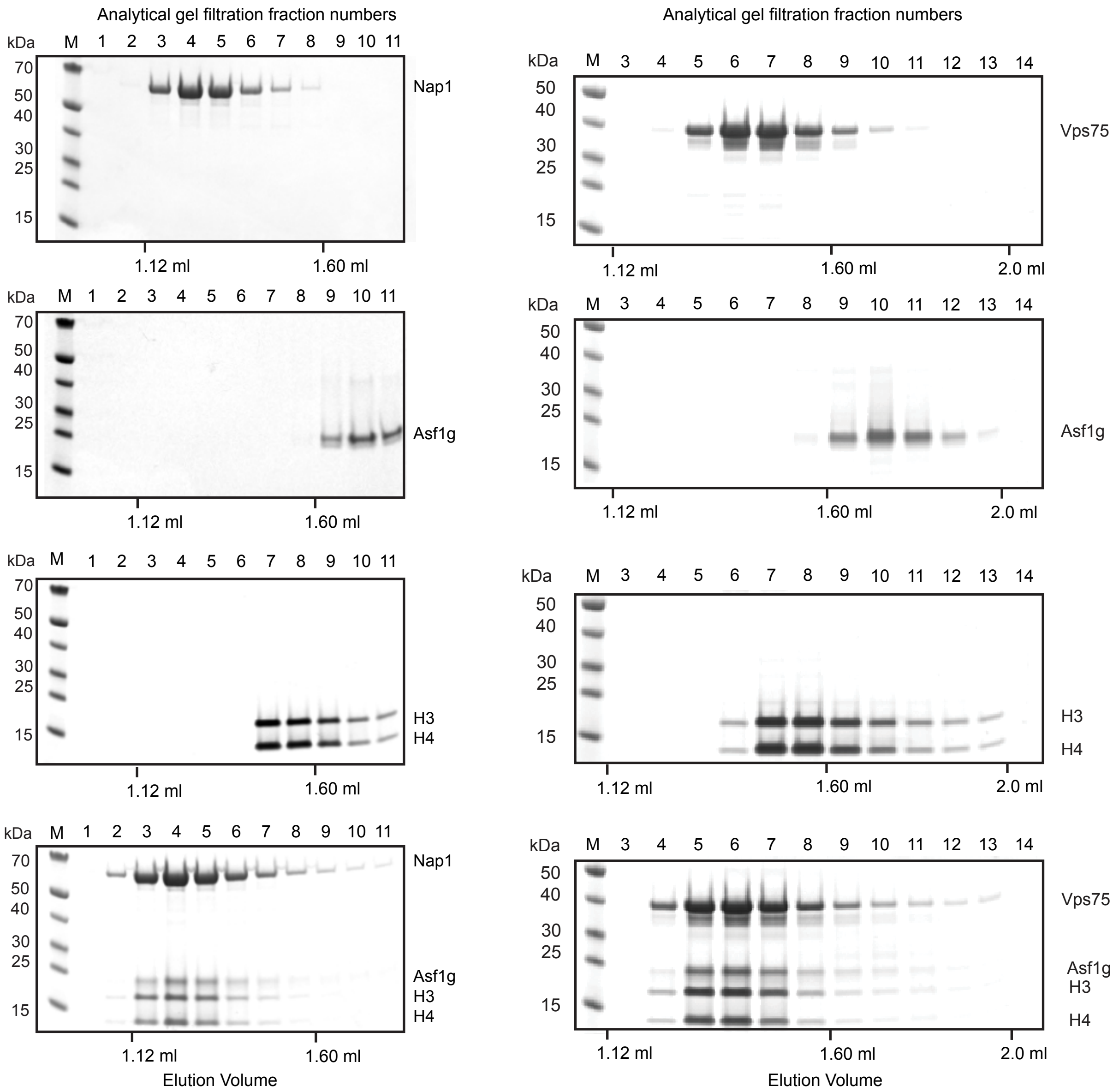
Analytical gel filtration fraction numbers
kDa
M
1 2 3 4 5 6 7 8 9 10 11
70
50
40
30
25
15
Nap1
1.12 ml
1.60 ml
Asf1g
H3
H4
Elution Volume
3 4 5 6 7 8 9 10 11 12 13 14
Vps75
2.0 ml

Figure S6

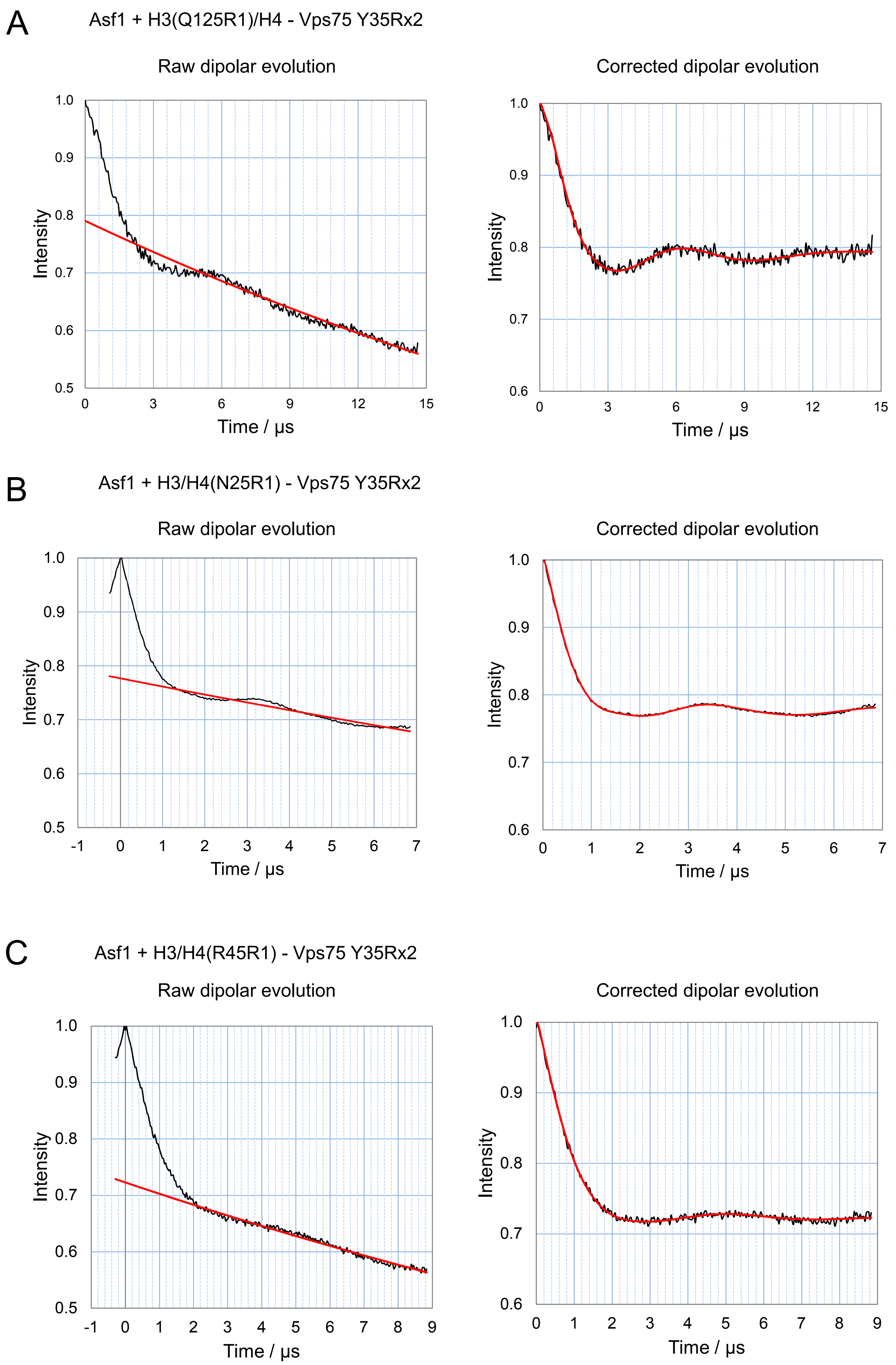
A
Asf1 + H3(Q125R1)/H4 - Vps75 Y35Rx2
Raw dipolar evolution
Intensity
Time / μs
Corrected dipolar evolution
Intensity
Time / μs
B
Asf1 + H3/H4(N25R1) - Vps75 Y35Rx2
Raw dipolar evolution
Intensity
Time / μs
Corrected dipolar evolution
Intensity
Time / μs
C
Asf1 + H3/H4(R45R1) - Vps75 Y35Rx2
Raw dipolar evolution
Intensity
Time / μs
Corrected dipolar evolution
Intensity
Time / μs

A

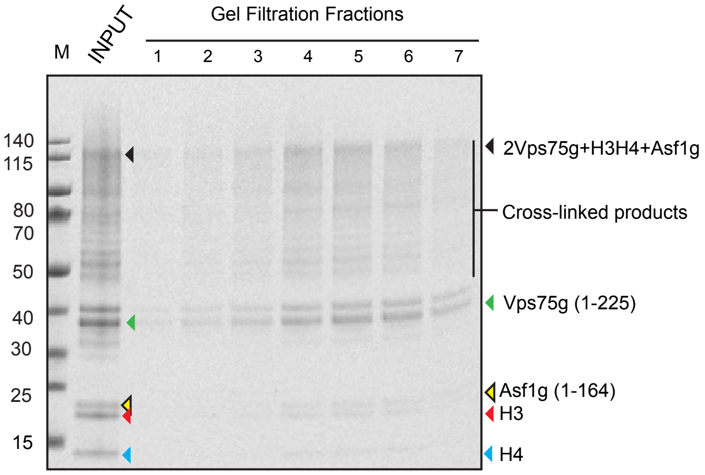

B

Vps75 K177 - Asf1 K143

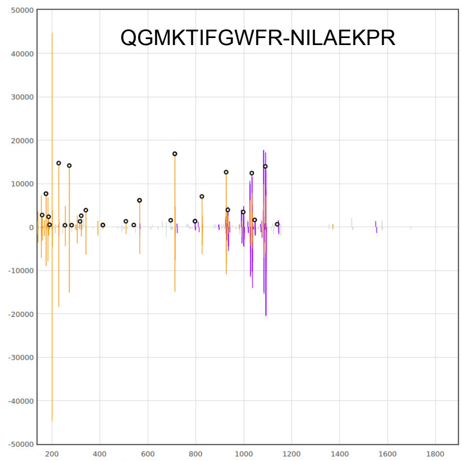

Yellow	Peaks in both spectra
Purple	d0/d4 BS2G shifted peaks between spectra
Black dot	Matched y and b-ions

alpha-chain (Vps75)

b-ion+1	129.07	186.09 (186.09)	317.13 (317.13)	445.22	546.27	659.36 (659.85)	806.42	863.44	1049.52	1196.59	1352.69
b-ion+2	65.04	93.55	159.07 (159.09)	223.12	273.64 (273.17)	330.18 (330.88)	403.72	432.23	525.27	598.80	676.85 (676.65)
b-ion+3	43.69	62.70	106.38	149.08	182.76 (183.11)	220.46	269.48	288.49	350.51	399.54	451.57
xlink-b-ion+1	1164.64	1221.66	1352.70	1480.79	1581.84	1694.93	1841.99	1899.02	2085.10	2232.16	2388.27
xlink-b-ion+2	582.82	611.33	676.85 (676.65)	740.90	791.43	847.97	921.50	950.01	1043.05	1116.59	1194.64
xlink-b-ion+3	388.88	407.89	451.57	494.27	527.95	565.65 (565.29)	614.67	633.68	695.70 (695.33)	744.73	796.76 (796.76)
AA	Q	G	M	K	T	I	F	G	W	F	R
y-ion+1		1242.65	1185.62	1054.58	926.49 (926.49)	825.44 (825.44)	712.36 (712.36)	565.29 (565.29)	508.27 (508.27)	322.19 (322.19)	175.12 (175.12)
y-ion+2		621.83	593.32	527.80	463.75	413.22 (412.26)	356.68	283.15 (282.18)	254.64 (254.15)	161.60	88.06
y-ion+3		414.89	395.88	352.20	309.50	275.82	238.12	189.10 (190.10)	170.09	108.07	59.05
xlink-y-ion+1		2278.22	2221.20	2090.16	1962.06	1861.01	1747.93	1600.86	1543.84	1357.76	1210.69
xlink-y-ion+2		1139.61 (1139.62)	1111.10	1045.58 (1045.58)	981.53 (981.51)	931.01	874.47	800.93	772.42	679.38	605.85
xlink-y-ion+3		760.08	741.07	697.39	654.69	621.01	583.31	534.29	515.28	453.26	404.24

beta-chain (Asf1)

b-ion+1	115.05	228.13 (228.13)	341.22 (341.22)	412.26 (412.26)	541.30 (541.24)	669.39	766.45	922.55
b-ion+2	58.03	114.57	171.11	206.63	271.15	335.20	383.73	461.78
b-ion+3	39.02	76.72	114.41	138.09	181.10	223.80	256.15	308.19
xlink-b-ion+1	1580.77	1693.85	1806.94	1877.97	2007.02	2135.11	2232.16	2388.27
xlink-b-ion+2	790.89	847.43	903.97	939.49	1004.01	1068.06	1116.59	1194.64
xlink-b-ion+3	527.59	565.29 (565.29)	602.98	626.66	669.68	712.38 (712.36)	744.73	796.76 (796.76)
AA	N	I	L	A	E	K	P	R
y-ion+1		826.52 (826.44)	713.43 (713.36)	600.35	529.31	400.27	272.17 (272.17)	175.12 (175.12)
y-ion+2		413.76	357.22	300.68 (300.10)	265.16	200.64 (200.14)	136.59	88.06
y-ion+3		276.18	238.48	200.79 (201.14)	177.11 (176.12)	134.09	91.40	59.05
xlink-y-ion+1		2292.23	2179.15	2066.06	1995.03	1865.98	1737.89	1640.84
xlink-y-ion+2		1146.62	1090.08 (1090.08)	1033.54 (1033.54)	998.02 (998.01)	933.50 (933.50)	869.45	820.92
xlink-y-ion+3		764.75	727.05	689.36	665.68	622.67	579.97	547.62 (548.26)

C

Vps75 K169 - Asf1 K143

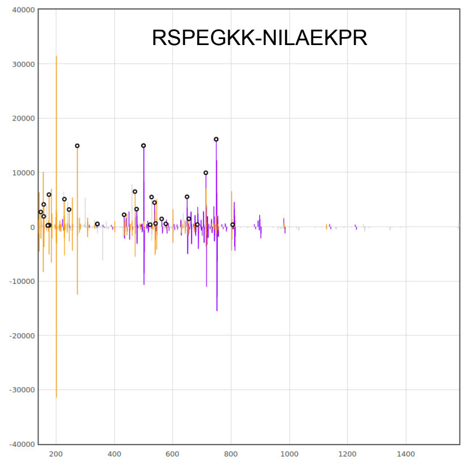

Yellow	Peaks in both spectra
Purple	d0/d4 BS2G shifted peaks between spectra
Black dot	Matched y and b-ions

alpha-chain (Vps75)

b-ion+1	157.11 (157.11)	244.14 (244.14)	341.19 (341.22)	470.24 (470.24)	527.26 (527.26)	655.35 (655.35)	783.45
b-ion+2	79.06	122.57	171.10 (171.11)	235.62	264.13	328.18	392.23
b-ion+3	53.04	82.05	114.40	157.42 (157.13)	176.42 (176.12)	219.12	261.82
xlink-b-ion+1	1192.68	1279.71	1376.77	1505.81	1562.83	1690.92	1819.02
xlink-b-ion+2	596.84	640.36 (640.36)	688.89	753.41 (753.90)	781.92	845.97	910.01
xlink-b-ion+3	398.23	427.24	459.59 (459.31)	502.61	521.61 (522.30)	564.31	607.01
AA	R	S	P	E	G	K	K
y-ion+1		645.36	558.33	461.27 (460.31)	332.23 (331.30)	275.21	147.11 (147.11)
y-ion+2		323.18	279.67 (279.17)	231.14 (230.15)	166.62 (167.06)	138.11	74.06
y-ion+3		215.79 (216.15)	186.78	154.43 (155.08)	111.42	92.41	49.71
xlink-y-ion+1		1680.93	1593.90	1496.84	1367.80	1310.78	1182.68
xlink-y-ion+2		840.97	797.45	748.93 (748.91)	684.40 (684.37)	655.89 (655.35)	591.85
xlink-y-ion+3		560.98 (561.82)	531.97	499.62 (499.61)	456.61	437.60	394.90

beta-chain (Asf1)

b-ion+1	115.05	228.13 (228.13)	341.22 (341.22)	412.26	541.30 (540.97)	669.39	766.45	922.55
b-ion+2	58.03	114.57	171.11 (171.11)	206.63	271.15	335.20 (336.16)	383.73	461.78
b-ion+3	39.02	76.72	114.41	138.09	181.10	223.80	256.15	308.19 (308.20)
xlink-b-ion+1	1011.52	1124.61	1237.69	1308.73	1437.77	1565.87	1662.92	1819.02
xlink-b-ion+2	506.27	562.81 (562.32)	619.35	654.87 (655.35)	719.39 (718.91)	783.44	831.96	910.01
xlink-b-ion+3	337.85	375.54	413.24	436.91	479.93	522.63 (522.30)	554.98	607.01
AA	N	I	L	A	E	K	P	R
y-ion+1		826.52	713.43 (713.43)	600.35 (600.35)	529.31 (529.31)	400.27 (400.27)	272.17 (272.17)	175.12 (175.12)
y-ion+2		413.76	357.22 (357.18)	300.68 (300.06)	265.16	200.64 (201.09)	136.59	88.06
y-ion+3		276.18	238.48 (239.09)	200.79 (201.09)	177.11 (176.12)	134.09	91.40	59.05
xlink-y-ion+1		1722.99	1609.90	1496.82	1425.78	1296.74	1168.64	1071.59
xlink-y-ion+2		862.00	805.46 (805.45)	748.91 (748.91)	713.39 (713.39)	648.87 (648.87)	584.83 (584.82)	536.30
xlink-y-ion+3		575.00 (575.82)	537.31 (537.30)	499.61 (499.61)	475.93 (475.93)	432.92 (432.92)	390.22	357.87 (357.18)

A Measuring distances from a singly labelled Vps75 dimer to a singly labelled H3H4 tetramer

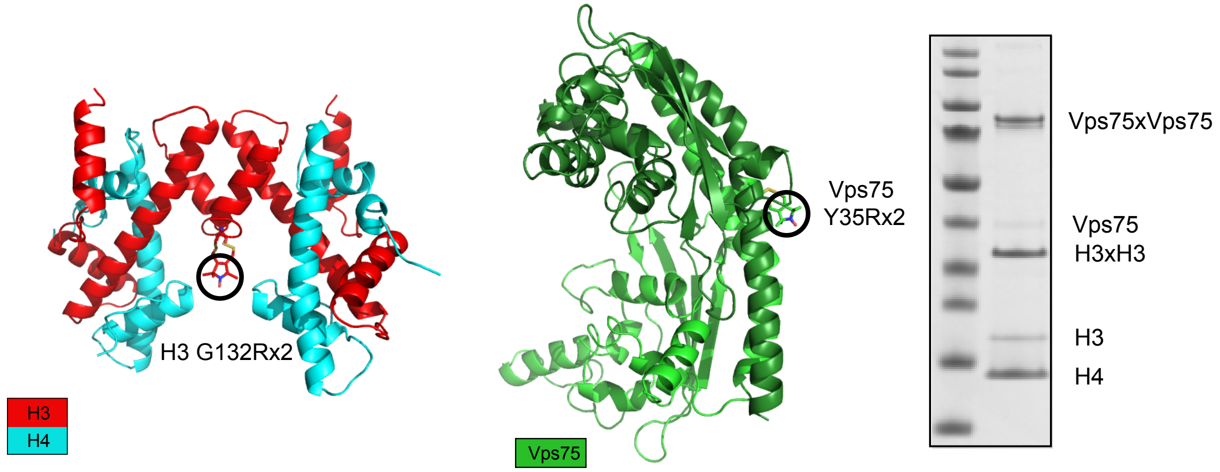

B $Vps75_2$ - $(H3H4)_2$ model

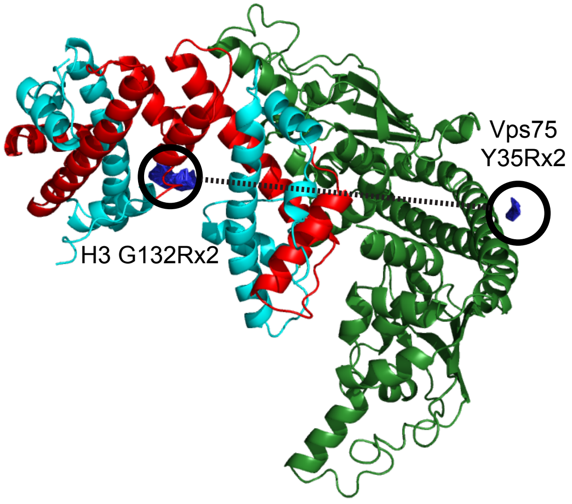

C Vps75 Y35Rx2 - H3 G132Rx2/H4 - 400 mM NaCl

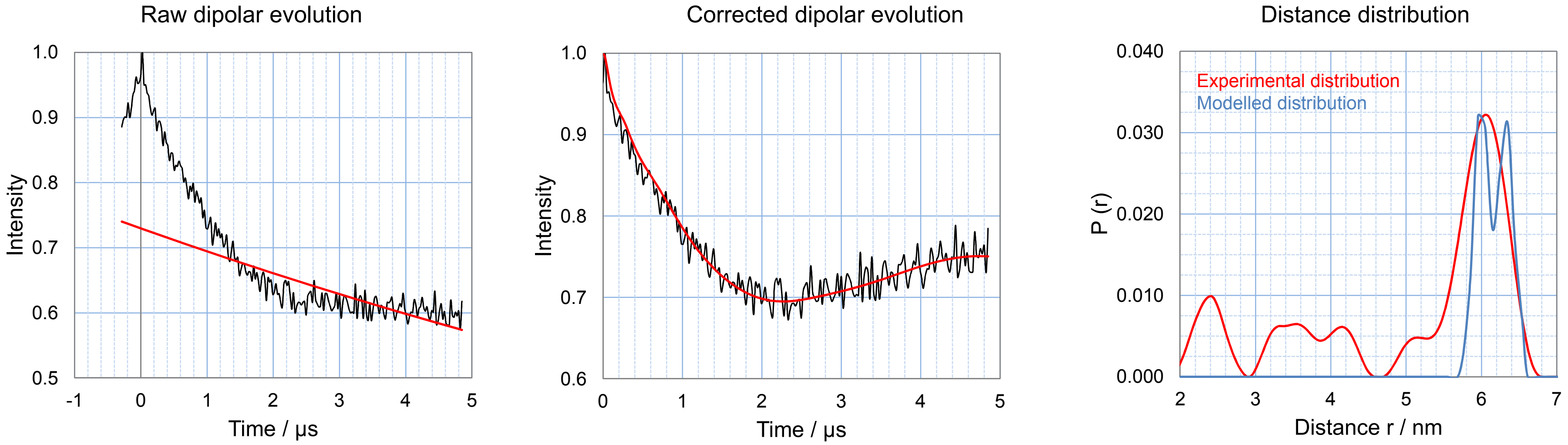

Table S1 Hammond et al 2016

Scan mode	Excitation	Emission
Start (nm)	260	325
Stop (nm)	360	550
Em. Wavelength (nm)	400	345
Ex. Slit (nm)	5	5
Em. Slit (nm)	5	5
Scan rate (nm/min)	120	120
Data interval (nm)	1	1
Averaging Time (s)	0.5	0.5
Excitation filter	Auto	Auto
Emission filter	Open	Open
PMT voltage (V)	Medium	Medium
Corrected spectra	OFF	OFF
Smoothing	ON	ON
Type	Savitzky-Golay	Savitzky-Golay
Filter size	5	5

Table S2 Hammond et al 2016

Number of observations inclusive of d0 and d4	Crosslink #	Protein (A)	Position (A)	Sequence (A)	Protein (B)	Position (B)	Sequence (B)	Score	PPM	FDR %
8	1	H3	9	KSTGGK	VpsdC	78	ASDFKYIDTIDK	650	0.571	0.00
12	2	H3	27	KSAPATGGVK	H4	91	TVTAMDVVYALKR	626	0.219	0.00
6	3	H4	31	VLRDNIQGITKPAIR	H4	5	GKGGK	610	0.344	5.00
6	4	H4	8	GGKGLGK	VpsdC	78	ASDFKYIDTIDK	599	0.283	4.35
4	5	H3	56	YQKSTELLIR	H3	64	KLPFQR	596	0.076	4.17
22	6	H3	18	KQLATK	H3	27	KSAPATGGVK	593	0.419	3.85
10	7	H3	18	KQLATK	H4	91	TVTAMDVVYALKR	556	0.965	3.23
4	8	H3	27	KSAPATGGVK	VpsdC	177	QGMKTIFGWFR	549	0.064	3.03
6	9	Asfg	143	NILAEKPR	VpsdC	177	QGMKTIFGWFR	527	0.807	2.70
12	10	H3	18	KQLATK	VpsdC	78	ASDFKYIDTIDK	513	0.087	2.63
6	11	H3	9	KSTGGK	H4	79	RKTVTAMDVVYALK	502	0.464	2.56
4	12	H3	18	KQLATK	VpsdC	177	QGMKTIFGWFR	498	0.864	2.38
8	13	Asfg	143	NILAEKPR	VpsdC	169	RSPEGKK	496	0.016	2.33
6	14	H3	14	STGGKAPR	VpsdC	78	ASDFKYIDTIDK	488	0.372	2.17
12	15	H3	18	KQLATK	VpsdC	177	YRQGMKTIFGWFR	485	0.494	2.08
10	16	H3	27	KSAPATGGVK	VpsdC	177	YRQGMKTIFGWFR	481	0.249	1.96
36	17	VpsdC	189	WTGLKPGK	VpsdC	163	DKRSPEGKK	477	0.534	1.92
10	18	H3	14	STGGKAPR	H4	91	TVTAMDVVYALKR	474	0.004	1.89
6	19	H3	18	KQLATK	H3	56	RYQKSTELLIR	473	0.85	1.85
8	20	H3	27	KSAPATGGVK	VpsdC	177	YRQGMKTIFGWFR	464	0.181	3.45
8	21	Asfg	143	NILAEKPR	H3	18	KQLATK	459	0.082	3.39

Hammond et a. 2016
Supplementary Table S3

Amino acid conservation of Vps75 residues involved in various protein-protein interactions.

Asymmetric_Vps75_Tetramer.pdb

Tetramerisation interface:

Vps75 Chain B		ConSurf Score	Vps75 Chain C		ConSurf Grade
GLN	64	7	SER	63	8
VAL	66	6	GLN	64	7
ASN	70	7	ALA	69	6
LYS	169	8	ARG	73	7
TYR	172	8	ALA	74	6
ARG	173	9	SER	75	6
MET	176	9	PHE	77	7
LYS	177	8	GLN	150	1
PRO	190	6	SER	151	6
GLY	191	7	SER	154	5
LYS	192	8	LYS	170	8
GLU	193	9	LYS	171	5
PHE	194	8	GLN	174	4
PRO	195	8	GLN	220	7
HIS	196	6	ARG	221	5
GLU	218	7	ASP	222	5
ARG	221	5	LEU	223	6
ASP	222	5	GLU	224	5
ASP	225	6	ASP	225	6
Interface size: 600 Å^2					

Symmetric_Vps75_Tetramer.pdb

Tetramerisation interface:

Vps75 Chain A		ConSurf Score	Vps75 Chain B		ConSurf Score
GLU	56	3	ILE	60	8
LYS	59	6	ALA	69	6
ALA	69	6	ASN	70	7
ASN	70	7	ILE	72	7
ALA	74	6	ARG	73	7
SER	75	6	ALA	74	6
PRO	166	1	PHE	77	7
LYS	169	8	LYS	170	8
LYS	170	8	ARG	173	9
LYS	173	9	GLN	220	7
LYS	177	8	ASP	222	5
GLY	191	7	LEU	223	6
ASP	222	5	GLU	224	5
LEU	223	6	ASP	225	6
GLU	224	5			
ASP	225	6			
Interface size: 314 Å^2					

VAH_average.pdb

Vps75 chain A interface with H3

Vps75 Chain A		ConSurf Score	H3	
ASP	133	5	ALA	60
GLN	134	1	LEU	61
GLU	135	4	ARG	63
PRO	166	1	LEU	65
LYS	169	8	GLU	94
ARG	173	9	GLU	97
PRO	190	6	VAL	101
GLY	191	7	ASN	108
HIS	196	6	ILE	112
ASP	198	8		
Interface size: 256.8 Å^2				

VAH_average.pdb

Vps75 chain A interface with H4

Vps75 Chain A		ConSurf Score	H4	
ARG	164	4	ARG	36
LYS	169	8	ARG	39
ARG	173	9	ARG	40
LYS	177	8	GLY	41
PRO	190	6	GLY	42
GLY	191	7	LYS	44
LYS	192	8		
GLU	193	9		
PRO	195	8		
Interface size: 264.1 Å^2				

VAH_average.pdb

Vps75 chain B interface with H3

Vps75 Chain B		ConSurf Score	H3	
GLN	64	7	LEU	65
VAL	66	6	GLN	68
TYR	215	7	ARG	69
GLU	218	7	ARG	72
ARG	221	5	GLU	73
ASP	222	5	ARG	83
ASP	225	6	PHE	84
			GLN	85
Interface size: 255.9 Å^2				

VAH_average.pdb

Vps75 chain B interface with H4

Vps75 Chain B		ConSurf Score	H4	
VAL	66	6	ARG	78
ALA	69	6	LYS	79
ASN	70	7	THR	80
Interface size: 91.0 Å^2				

Vps75-H3H4 total interface size: 869.8 Å2

* Residue conservation calulated using ConSurf and an alignemnet of Vps75 protein sequence from against homologues from Fungi as stated in Supplemental document
* Residues are stated in numerical order and pairwise interactions should not be conferred from placement of residues within each table

pos	MAX AA	ConSurf Score	pos	MAX AA	ConSurf Score
1	M 86.364	7	133	D 58.621	5
2	M 88.608	8	134	Q 41.860	1
3	S 54.545	4	135	E 68.182	4
4	D 34.426	1	136	D 70.455	2
5	Q 37.500	1	137	G 52.500	3
6	E 54.545	1	138	I 40.000	2
7	N 81.818	6	139	L 90.476	7
8	E 58.824	1	140	T 57.143	6
9	N 65.714	4	141	S 100.000	9
10	E 65.714	1	142	E 66.667	4
11	H 48.485	1	143	P 63.636	6
12	A 54.545	3	144	V 83.838	8
13	K 66.667	2	145	E 37.374	2
14	A 51.190	6	146	I 61.616	7
15	L 54.545	7	147	E 42.424	2
16	L 30.682	2	148	W 93.939	7
17	G 27.273	1	149	P 75.269	9
18	L 94.565	9	150	K 29.032	1
19	A 65.217	7	151	S 40.217	6
20	D 35.870	3	152	Y 39.796	7
21	C 43.478	4	153	D 47.475	5
22	E 95.652	9	154	S 41.463	5
23	E 21.739	1	155	I 70.707	8
24	E 67.391	4	156	N 69.697	8
25	M 36.170	5	157	P 71.429	5
26	D 34.043	4	158	D 31.111	1
27	K 30.851	2	159	L 44.944	1
28	V 32.979	6	160	I 81.013	8
29	E 92.473	9	161	K 63.291	4
30	R 41.935	3	162	D 50.633	4
31	E 64.516	5	163	K 57.353	6
32	V 37.634	5	164	R 32.857	4
33	E 69.149	7	165	S 72.222	7
34	I 30.108	4	166	P 37.975	1
35	Y 61.290	2	167	E 55.844	4
36	R 74.194	8	168	G 49.351	4
37	L 40.860	4	169	K 92.308	8
38	K 46.237	3	170	K 89.744	8
39	K 54.839	1	171	N 46.154	5
40	T 33.333	5	172	Y 92.405	8
41	K 33.673	3	173	R 94.937	9
42	P 80.612	6	174	Q 40.506	4
43	V 34.694	6	175	G 93.671	9
44	Y 86.735	8	176	M 90.000	9
45	E 39.796	1	177	K 90.123	8
46	K 69.388	5	178	S 59.000	8
47	R 100.000	9	179	F 74.000	8
48	D 39.796	6	180	F 98.000	9
49	A 26.531	1	181	G 30.000	6
50	I 43.878	5	182	W 85.000	4
51	I 47.959	7	183	F 97.000	9
52	K 36.735	3	184	R 32.323	2
53	E 31.633	1	185	W 92.929	6
54	I 90.816	8	186	T 86.869	9
55	P 63.265	6	187	G 98.990	9
56	K 28.571	3	188	L 41.860	3
57	F 98.980	9	189	K 82.558	7
58	W 100.000	9	190	P 81.395	6
59	K 33.673	6	191	G 87.209	7
60	I 77.551	8	192	K 83.721	8
61	V 87.755	8	193	E 93.023	9
62	L 90.816	8	194	F 95.455	8
63	S 41.837	8	195	P 52.273	8
64	Q 52.041	7	196	N 45.455	6
65	H 50.515	8	197	G 98.980	9
66	D 42.857	6	198	D 54.082	8
67	D 47.959	6	199	S 30.612	6
68	F 89.130	8	200	L 86.735	6
69	A 64.286	6	201	A 84.694	8
70	N 41.837	7	202	S 22.449	4
71	Y 88.776	7	203	L 78.571	6
72	I 73.469	7	204	F 42.268	5
73	R 54.082	7	205	S 46.392	5
74	A 48.980	6	206	E 73.958	6
75	S 37.755	6	207	D 59.794	8
76	D 100.000	9	208	L 49.485	6
77	F 41.837	7	209	Y 68.041	6
78	K 74.490	7	210	P 78.351	8
79	Y 74.490	7	211	N 35.052	6
80	L 42.857	5	212	A 51.546	9
81	E 52.041	7	213	V 68.041	8
82	A 21.429	2	214	K 86.598	8
83	I 81.633	8	215	Y 89.691	7
84	D 38.776	1	216	Y 84.536	8
85	D 39.796	6	217	T 46.392	8
86	I 68.367	7	218	E 65.979	7
87	Y 38.144	4	219	A 90.722	9
88	V 95.876	9	220	Q 48.421	7
89	E 37.113	2	221	R 43.750	5
90	W 44.330	7	222	D 52.577	5
91	L 32.292	1	223	L 35.632	6
92	I 24.731	3	224	E 64.368	5
93	L 36.250	1	225	D 70.968	6
94	E 39.506	2	226	E 63.736	6
95	DS 27.059	1	227	E 49.451	4
96	E 47.273	1	228	E 37.037	4
97	M 27.273	1	229	E 67.470	5
98	A 32.759	1	230	S 60.000	6
99	D 54.717	1	231	G 37.500	6
100	P 48.333	6	232	E 46.154	6
101	R 66.279	7	233	G 49.020	7
102	D 77.320	6	234	E 62.745	8
103	F 82.653	7	235	E 55.172	6
104	S 42.857	5	236	L 43.590	5
105	I 85.714	8	237	D 88.462	8
106	T 79.592	7	238	L 49.351	5
107	F 41.837	5	239	S 44.304	7
108	T 23.469	3	240	D 59.259	4
109	F 97.959	9	241	D 68.354	5
110	H 23.232	1	242	D 53.846	5
111	G 29.293	1	243	G 32.877	4
112	I 40.964	5	244	S 39.326	5
113	E 45.000	1	245	E 48.000	4

Consurf Score	
9	Conserved
8	
7	
6	
5	Average
4	
3	
2	
1	Variable

114	G	40.541	5	246	G	32.836	1
115	D	41.758	3	247	E	62.162	4
116	F	39.796	6	248	L	44.444	2
117	P	37.374	2	249	D	40.909	3
118	E	45.455	2	250	L	38.750	2
119	Q	81.818	9	251	P	33.333	2
120	T	23.469	4	252	L	30.435	1
121	V	54.545	6	253	S	34.286	1
122	T	60.606	7	254	D	37.681	3
123	K	98.990	9	255	E	53.968	2
124	V	25.510	2	256	E	64.407	1
125	F	93.878	9	257	P	40.000	1
126	K	29.293	1	258	S	33.333	3
127	I	47.475	2	259	S	48.000	2
128	K	27.368	1	260	K	97.260	9
129	K	35.632	3	261	K	78.082	6
130	G	29.545	1	262	R	38.356	3
131	K	37.037	1	263	K	76.389	6
132	D	68.966	5	264	V	42.308	2
